# Priming in the Type I-F CRISPR-Cas system triggers strand-independent spacer acquisition, bi-directionally from the primed protospacer

**DOI:** 10.1093/nar/gku527

**Published:** 2014-07-01

**Authors:** Corinna Richter, Ron L. Dy, Rebecca E. McKenzie, Bridget N.J. Watson, Corinda Taylor, James T. Chang, Matthew B. McNeil, Raymond H.J. Staals, Peter C. Fineran

**Affiliations:** Department of Microbiology and Immunology, University of Otago, PO Box 56, Dunedin 9054, New Zealand

## Abstract

Clustered regularly interspaced short palindromic repeats (CRISPR), in combination with CRISPR associated (*cas*) genes, constitute CRISPR-Cas bacterial adaptive immune systems. To generate immunity, these systems acquire short sequences of nucleic acids from foreign invaders and incorporate these into their CRISPR arrays as spacers. This adaptation process is the least characterized step in CRISPR-Cas immunity. Here, we used *Pectobacterium atrosepticum* to investigate adaptation in Type I-F CRISPR-Cas systems. Pre-existing spacers that matched plasmids stimulated hyperactive primed acquisition and resulted in the incorporation of up to nine new spacers across all three native CRISPR arrays. Endogenous expression of the *cas* genes was sufficient, yet required, for priming. The new spacers inhibited conjugation and transformation, and interference was enhanced with increasing numbers of new spacers. We analyzed ∼350 new spacers acquired in priming events and identified a 5′-protospacer-GG-3′ protospacer adjacent motif. In contrast to priming in Type I-E systems, new spacers matched either plasmid strand and a biased distribution, including clustering near the primed protospacer, suggested a bi-directional translocation model for the Cas1:Cas2–3 adaptation machinery. Taken together these results indicate priming adaptation occurs in different CRISPR-Cas systems, that it can be highly active in wild-type strains and that the underlying mechanisms vary.

## INTRODUCTION

CRISPR-Cas (clustered regularly interspaced short palindromic repeats-CRISPR-associated proteins) is a small RNA-based prokaryotic defense mechanism that functions against a wide range of mobile genetic elements (MGEs) (for recent reviews see (1–6)). Immunity against MGEs is provided through a sequence-specific adaptive memory. CRISPR arrays are characterized by short repeats separated by similarly sized sequences (spacers), which are derived from invading MGEs. The mechanism of CRISPR interference involves three phases. During adaptation, short sequences derived from invader nucleic acids are incorporated as new spacers into the CRISPR array. Next, the expression stage results in the transcription of the CRISPRs from a promoter in a leader sequence upstream of the CRISPR arrays. These pre-crRNAs are processed into small RNAs (crRNAs) by endonucleolytic Cas proteins. Finally, in the interference step, Cas protein(s) and crRNAs form ribonucleoprotein complexes that target and degrade invading nucleic acids, which possess complementarity to the spacer portion of the crRNA. CRISPR-Cas systems are currently classified into three major Types (I–III), which are characterized by signature proteins (Cas3, Cas9 and Cas10, respectively) and further subdivided into subtypes, based on subtype-specific proteins (7). Considerable advances in understanding the expression and interference phases in different CRISPR-Cas systems have been made. In contrast, despite some knowledge about the adaptation phase, particularly in the Type I-E system (8–15), little is known about adaptation in the Type I-F systems (reviewed in (16)).

The acquisition of spacers upon an encounter with new foreign genetic elements is the prerequisite for developing CRISPR-Cas resistance against this invader. This was first observed for the Type II system of *Streptococcus thermophilus*, where new phage-derived spacers were detected in CRISPR arrays following the isolation of phage resistant strains (17). Spacers were acquired at the leader end of the CRISPR array (17) and this has been observed in subsequent experiments in other systems (12,18–20). Unusually, in *Sulfolobus solfataricus* P2, internal spacer acquisition was observed specifically in one of the six CRISPR loci (19). Importantly, adaptation can occur through two different pathways, termed naïve and priming, which will be discussed below (16).

Genetic experiments in *Escherichia coli* have begun to provide insight into the process of CRISPR adaptation in the Type I-E system (8–15). Only Cas1 and Cas2 are conserved among all CRISPR-Cas types and are required for new spacer incorporation in the Type I-E system (8,9,12). Recognition, and presumably cleavage, of the invader genome to generate new spacers involves detection of short motifs adjacent to the precursor spacer, which are termed protospacer adjacent motifs (PAMs; or spacer acquisition motifs) (9,12,21,22). Protospacers are the targeted/complementary strand of crRNAs and are denoted in the 5′-3′ direction (5,23)). The new spacer is then integrated at the leader-proximal end of the CRISPR, resulting in duplication of the first repeat (8,11,12).

In the arms race between MGEs and their hosts, the invaders can ‘escape’ CRISPR-Cas immunity with point mutations that disrupt target recognition (8,24). Surprisingly, recent studies in Type I-E systems revealed that a second adaptation stage, termed priming, enables rapid adaptation to these escapees (8,10,11,15). In priming, invading escape MGEs are not immediately targeted, but trigger the accelerated incorporation of new spacers, ultimately resulting in interference (8,11). Multiple spacers can be incorporated, providing increased resistance and reducing the probability of further escape (11). In contrast to naïve adaptation, where Cas1 and Cas2 are the only Cas proteins required for spacer integration in Type I-E systems (12), priming needs Cas1, Cas2, Cas3, crRNA and Cascade (CRISPR-associated complex for antiviral defense) (8). In Type II-A and II-B systems, in addition to Cas1 and Cas2, Csn2 is required (17), and Cas4 is predicted to be involved, in the acquisition process (7). Priming in *E. coli* results in a spacer strand bias, whereby new spacers target the same strand as the original priming spacer, but the underlying mechanism is unknown (8,10,11,15). In contrast, priming was recently observed in the Type I-B system of *Haloarcula hispanica*, but new spacers targeted either strand of the invader DNA (20). Recently, we discovered that adaptation in the Type I-E system is primed by pre-existing spacers, even when they mismatch the invader by as much as 13 nt across the 35 nt PAM-protospacer sequence (15). A recent analysis of the PAM requirements for priming in a Type I-B system (25) confirmed our results that multiple PAMs can promote priming (15). This demonstrates that CRISPR-Cas systems are robust in their ability to restore resistance to escaping invaders, and suggests that resistance is generated more easily against closely related genetic elements that have not been previously encountered (15). This supports the concept that priming is the major contributor to adaptation when compared to naïve acquisition.

In contrast to the Type I-E system, there is limited knowledge about acquisition in Type I-F systems. With the exception of a report where a few single spacers (seven unique) were incorporated (18), no information is available about adaptation in Type I-F systems. The I-F subtype is unique for studying acquisition as it is the only system in which the universal Cas2 and the signature Cas3 protein are fused as a hybrid-protein (Cas2–3) (7,26). We previously demonstrated that Cas2–3 interacts with Cas1, suggesting that a Cas1:Cas2–3 adaptation complex engages in spacer incorporation (26). Here, to investigate acquisition in the Type I-F systems, we used the potato pathogen *Pectobacterium atrosepticum* (27). We have previously shown that this strain contains a single Type I-F *cas* operon and three CRISPR arrays that are transcribed and processed by Cas6f (Csy4) to yield crRNAs (28). The Csy1, Csy2, Csy3 and Cas6f proteins form an interference complex (26,29) that can interact with Cas2–3 (26). In this study, we detected hyperactive priming adaptation, with up to nine new spacers in a single strain and all three CRISPR arrays were active for incorporation, albeit with different efficiencies. Type I-F priming resulted in a biased spacer acquisition pattern with new spacers complementary to both strands. The protospacer distribution supports a translocation model of localized spacer acquisition in Type I-F systems that is distinct from the strand-specific mechanism utilized by the Type I-E systems.

## MATERIALS AND METHODS

### Bacterial strains and growth conditions

All strains and plasmids used in this study are given in Supplementary Table S1 and details of their construction provided in Supplementary Materials and Methods. *P. atrosepticum* SCRI1043 (30) was grown at 25°C and *E. coli* at 37°C in Luria Broth (LB) at 180 rpm or on LB-agar (LBA) plates containing 1.5% (w/v) agar. When required, media were supplemented with the following: ampicillin (Ap; 100 μg/ml), chloramphenicol (Cm; 25 μg/ml), kanamycin (Km; 50 μg/ml), tetracycline (Tc; 10 μg/ ml) and D-glucose (0.2% w/v). Bacterial growth was measured in a Jenway 6300 spectrophotometer at 600 nm (OD_600_).

### Molecular biology and DNA sequencing

Oligonucleotides were from Invitrogen or IDT and are listed in Supplementary Table S2. All strains and plasmids were confirmed by polymerase chain reaction (PCR) and DNA sequencing was performed at the Allan Wilson Centre, New Zealand. Plasmid DNA was prepared using Zyppy Plasmid Miniprep Kits (Zymo Research). DNA from PCR and agarose gels was purified using the GE Healthcare Illustra GFX PCR DNA and Gel Band Purification Kit. Restriction enzymes and T4 DNA ligase were from Roche or NEB.

### Priming assays

**Figure 1.**
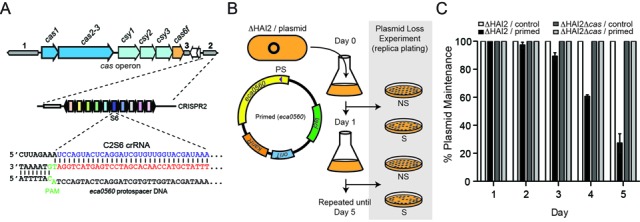
A pre-existing spacer:protospacer match accelerates Cas-dependent plasmid loss. (**A**) *P. atrosepticum* contains a Type I-F CRISPR-Cas system composed of three CRISPR arrays (1–3; gray arrows) and an operon of 6 *cas* genes (colored arrows). CRISPR2 consists of 10 spacers and spacer 6 (from leader proximal end; blue) perfectly matches a protospacer (red) in *eca0560* in the chromosomal island HAI2, but has a TG PAM variant. (**B**) Schematic of the plasmid loss assays. *P. atrosepticum* ΔHAI2 carrying pTRB30 (control) or pPF189 (*eca0560* primed; depicted) plasmids were grown without selection for 5 days and plasmid loss was scored by replica-plating on non-selective (NS) and selective (S) media. (**C**) Plasmid loss of a control plasmid (pTRB30) and the primed plasmid (pPF189) over 5 days when cultured in ΔHAI2 or ΔHAI2Δ*cas* backgrounds. Data shown are the mean ± SD of three biological replicates.

Five millilitres cultures of *P. atrosepticum* ΔHAI2 with pTRB30 (vector control) or pPF189 were grown overnight without antibiotic selection (Figure 1). Note that 10 μl were used to inoculate a fresh overnight culture and dilutions were plated onto LBA. This was repeated over 5 days and performed in triplicate. Colonies (100) from each replicate were patched onto LBA ± Km. Km sensitive (Km^S^) colonies were screened by PCR for new spacers as described later.

*P. atrosepticum* containing plasmids pPF571 (vector control), pPF574 and pPF575 (priming vectors with protospacer 1 from the CRISPR1 array with a mutated PAM in F and R orientations) were grown overnight in 5 ml LB and passaged for 5 days by transfer of 10 μl to 5 ml fresh LB. Additionally, dilutions were plated on LBA + 1 mM Isopropyl β-D-1-thiogalactopyranoside (IPTG) for mCherry induction. White colonies were screened for CRISPR expansion and sequenced as outlined below.

### CRISPR PCR and sequence analysis

Colonies displaying plasmid loss were screened by colony PCR using primers for CRISPR1 (PF174 and PF175), CRISPR2 (PF176 and PF177) or CRISPR3 (PF178 and PF179). The resulting products were separated on 2% agarose gels, purified and sequenced using PF175, PF177 or PF179 for CRISPR arrays 1, 2 or 3, respectively. Sequences were analyzed using CRISPRFinder (31), spacer sequences were extracted and assembled against target plasmids using Geneious™ and CRISPRTarget (23) to define the protospacer location, target strand and PAM.

### Transformation and conjugation assays

Electrocompetent *P. atrosepticum* cells were prepared as described previously (32). For transformations, 50 ng of DNA was added to 50 μl of competent cells, incubated on ice for 10 min then electroporated (1 mm electro-cuvettes, 1800 V, capacitance 25 μF and resistance 200 ohms). Bacteria were recovered in 1 ml LB for 2 h at 25°C and then plated on LB containing the appropriate supplements and grown at 25°C. Transformation efficiency was calculated as transformants/ng of DNA and normalized to non-targeted plasmid controls.

For conjugation, the tested plasmids were transformed into *E. coli* S17–1 λpir. Donor (*E. coli* S17–1 λpir with tested plasmids) and recipient strains were grown overnight in LB with the appropriate antibiotics. The OD_600_ was adjusted to 1 and cells washed twice with LB. The donor and recipient strains were mixed (1:1 ratio), 5 μl of the mixture spotted on 0.2 μm filters (Millipore) on LBA and incubated overnight. Cells were resuspended in 2 ml phosphate buffered saline by vortexing the filters and dilution series were plated on LBA (total cells), LBA + Sp (donors) and glucose minimal medium + Km (transconjugants). The efficiency of conjugation was calculated as transconjugants per recipients.

## RESULTS

### A pre-existing spacer:protospacer match accelerates Cas-dependent plasmid loss

To test whether priming was a general CRISPR phenomenon, we examined if the Type I-F system could acquire new spacers from a foreign element when an existing spacer was present. Previously, we have shown that *P. atrosepticum* contains a native spacer within CRISPR2 (termed C2S6; CRISPR2 Spacer 6) that has perfect complementarity to a chromosomal gene (*eca0560*) inside an integrative and conjugative element termed horizontally acquired island 2 (HAI2) (28). We previously demonstrated that this spacer was non-functional for interference due to a variant PAM (5′-protospacer-TG-3′) (32,33) (Figure 1A). The lack of interference with C2S6 led us to query whether this -1 PAM point mutation would trigger priming.

To test whether a native spacer in the chromosomal Type I-F array could promote spacer acquisition, we used strain ΔHAI2 (to prevent self-targeting due to priming), in which HAI2, including *eca0560*, was fully deleted, and introduced a control plasmid or a plasmid containing *eca0560*. Cultures were grown for 5 days and plasmid loss was determined (Figure 1B and C). The control plasmid was stable, whereas the plasmid with the protospacer (*eca0560*) was progressively lost over the time course, with 75% loss by day 5 (Figure 1C). To test the contribution of the Cas proteins, the experiment was also performed in an isogenic *cas* operon deletion strain (ΔHAI2Δ*cas*). No plasmid loss was detected in the absence of the *cas* genes (Figure 1C), confirming that plasmid curing driven by a pre-existing spacer:protospacer match was a Cas-dependent process.

### Plasmid loss is accompanied by spacer incorporation in all CRISPR arrays

**Figure 2.**
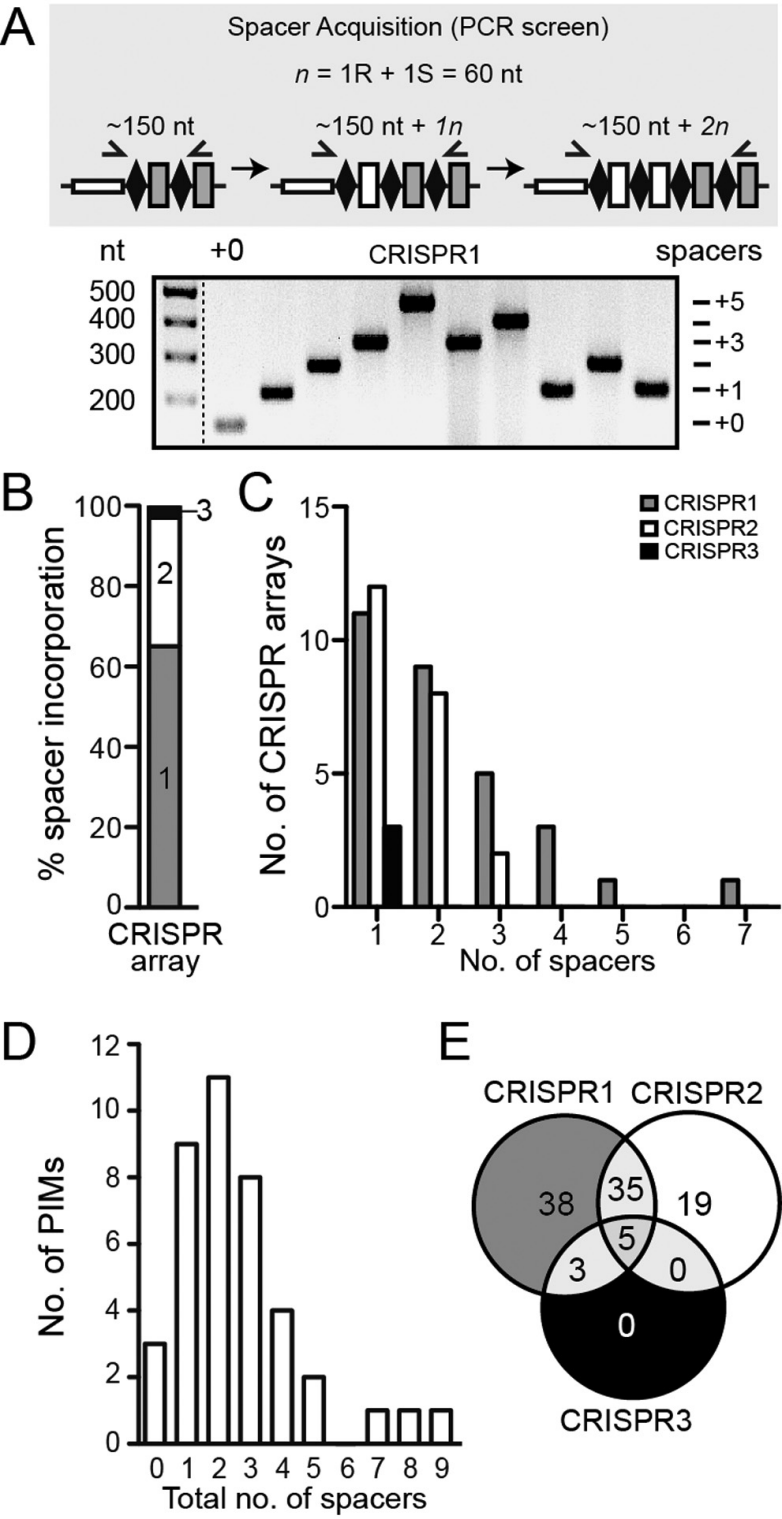
Plasmid loss is accompanied by spacer incorporation in all CRISPR arrays. (**A**) Schematic of the spacer acquisition PCR screen (R, repeat and S, spacer). Forward primers specific to each CRISPR array (1–3) were used with reverse primers specific to the second spacer in each CRISPR and products separated on a gel (example shown). (**B**) Percentage distribution of the total number of new spacers in each CRISPR array. (**C**) Number of CRISPR arrays containing a particular number of new spacers. (**D**) Number of PIMs containing a total number of new spacers across all CRISPR arrays. (**E**) Percentage distribution of PIMs containing additional spacers in particular CRISPR arrays (e.g. 35% of PIMs contain new spacers only in both CRISPR1 and 2).

To confirm that plasmid loss was CRISPR-dependent, expansion of the three native CRISPR arrays (CRISPR1, 2 and 3) was assessed (Figure 2A). Forty plasmid interfering mutants (PIMs) from independent experiments (to reduce siblings) from days 2 and 3 were checked by PCR, and 37/40 (93%) had at least one new spacer (Supplementary Table S3), whereas ∼7% were the result of CRISPR-independent plasmid loss. The CRISPR arrays of 37 CRISPR-dependent PIMs were sequenced and the acquired spacers were analyzed, revealing 105 new spacers (Supplementary Table S3). Sequencing the arrays revealed that all of the PIMs were unique and there were 68 spacers in CRISPR1 (65%), 34 spacers in CRISPR2 (32%) and 3 spacers in CRISPR3 (<3%) (Figure 2B). When analyzed for incorporation activity per array in each PIM, one acquisition event was the most common and fewer PIMs were observed with increasing numbers of newly-acquired spacers per array (Figure 2C). Notably, CRISPR1 and CRISPR2 had a similar activity for acquiring the first and second spacers, but CRISPR1 was more active for incorporating multiple spacers and only single, independent incorporations occurred in CRISPR3 (Figure 2C). There was considerable variation in the total number of spacers acquired across all CRISPR arrays by each strain, with 2 spacers the most frequent number incorporated (Figure 2D). Remarkably, one strain (PIM18) incorporated a total of 9 new spacers, 7 in CRISPR1 and 2 in CRISPR2 (Figure 2D and Supplementary Table S3). Of the PIMs with new spacers, most (81%) have at least one spacer in CRISPR1, 59% have at least one spacer in CRISPR2 and 8% have a spacer in CRISPR3 (Figure 2E). The array activity per PIM is outlined in Figure 2E (i.e. 38% of PIMs have new spacers only in CRISPR1, yet 35% have new spacers in both CRISPR1 and CRISPR2). This demonstrates that all arrays are proficient for integration and suggests that the order of incorporation activity of these arrays is CRISPR1 > CRISPR2 > CRISPR3. These data are highly consistent with the number of spacers present in the wild type (WT) strain (28, 10 and 3, respectively (27)) (Supplementary Figure S1) and promoter expression data, which showed a similar trend (28). The different spacer acquisition efficiencies might be the result of repeat or leader variations. The consensus sequence of the CRISPR1 and CRISPR2 repeats are the same, whereas the CRISPR3 repeats differ slightly (27). In addition, the leaders of each array are distinct, despite sharing some regions in common (data not shown). In conclusion, enhanced acquisition of nascent spacers occurs in native Type I-F systems (i.e. without *cas* gene overexpression) when a pre-existing interference-deficient spacer matches an MGE, but lacks a consensus PAM (see below).

### New spacers inhibit transformation and conjugation

**Figure 3.**
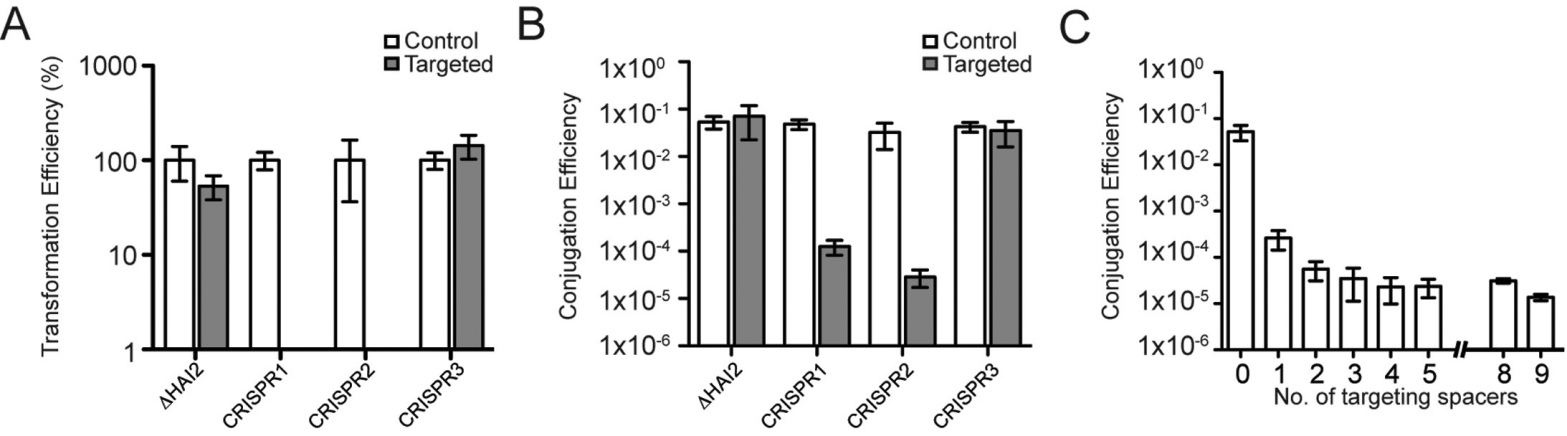
New spacers inhibit transformation and conjugation. (**A**). Transformation efficiency of strains with either no new spacers (ΔHAI2) or a single new spacer in CRISPR1 (PIM06), CRISPR2 (PIM17) or CRISPR3 (PIM23). Transformation was performed with a non-targeted control (pTRB30) or a targeted plasmid (pPF189 for CRISPR1 and 2 or pPF511 for CRISPR3). Efficiency was calculated as cfu/μg DNA and represented as % relative to the control vector (pTRB30). (**B**) Conjugation efficiency of strains with either no new spacers (ΔHAI2) or a single new spacer in CRISPR1 (PIM06), CRISPR2 (PIM17) or CRISPR3 (PIM23). Conjugation was performed with a non-targeted control (pPF260) or a targeted plasmid (pPF142 for CRISPR1 and 2 or pPF641 for CRISPR3). (**C**) Conjugation efficiency of plasmid pPF142 (*eca0560*) into strains with either no new spacers (ΔHAI2) or one (PIM06), two (PIM19), three (PIM13), four (PIM20), five (PIM32), eight (PIM30) or nine (PIM18) new spacers targeting pPF142. Data shown are the mean ± SD of three biological replicates.

We hypothesized that the acquired spacers caused plasmid loss. However, plasmid interference in the *P. atrosepticum* Type I-F system has not been demonstrated. To confirm the spacers acquired were responsible for plasmid loss, we tested the transformation efficiency of PIMs containing a single spacer in CRISPR1 or CRISPR2 that targeted *eca0560*. Control or targeted (*eca0560*) plasmids were used. No PIM contained a new spacer exclusively in CRISPR3. Therefore, we transformed PIM23, which contains single new spacers in both CRISPR1 and CRISPR3, with a plasmid containing the protospacer targeted by the new CRISPR3 spacer, but lacking the protospacer for the new CRISPR1 spacer. The ΔHAI2 strain, which contained no spacers targeting the plasmids, and the different PIMs were transformed with the targeted and non-targeted plasmids. The presence of a single additional spacer in CRISPR1 or CRISPR2 was sufficient to reduce transformation efficiency by >100-fold (the limit of detection) (Figure 3A). Surprisingly, the CRISPR3 spacer did not protect PIM23 from plasmid uptake (Figure 3A). A subtle difference in transformation efficiency was observed between the control and targeted plasmids in the non-targeting ΔHAI2 control strain, which is likely due to differences in plasmid size. However, these minor differences cannot account for the level of plasmid interference observed in the PIMs.

Next, we tested if new spacers inhibited conjugation. A single spacer in either CRISPR1 or CRISPR2 caused between a 350- and 1100-fold reduction in conjugation (Figure 3B), providing the first evidence of inhibition of conjugation by Type I-F systems. In agreement with the transformation result, the CRISPR3 spacer provided no protection from conjugation (Figure 3B). Multiple spacer acquisitions were observed during priming (Figure 2); therefore, we used the conjugation assays to test if multiple spacers led to elevated plasmid interference. We tested PIMs containing different numbers of spacers in the active arrays (CRISPR1 and 2) and that had protospacer targets with consensus PAMs (see below). Increasing the number of spacers further reduced the conjugation efficiency and the highest protection, of ∼3400-fold, was observed with nine new spacers (Figure 3C and Supplementary Figure S2). On average, across all PIMs tested, each spacer provided ∼400-fold protection, but not all spacers provided equal protection. For example, PIMs with 4 or 5 spacers provided similar interference (Supplementary Figure S2), which might be due to factors such as altered crRNA abundance or stability, resulting in differences in targeting. In summary, the new spacers acquired by the Type I-F system resulted in plasmid curing, inhibited both transformation and conjugation and additional spacers increased the level of protection.

### Protospacer distributions reveal biased spacer incorporation

**Figure 4.**
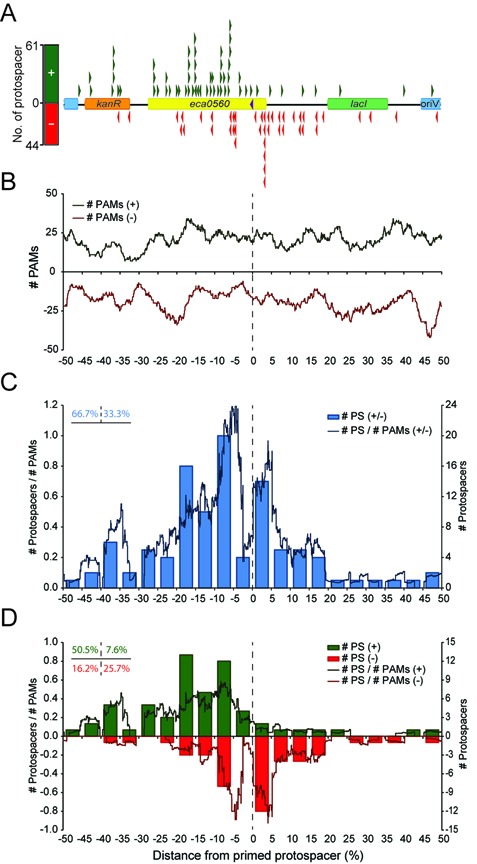
Protospacer distributions reveal biased spacer incorporation. (**A**) Linear representation of the *eca0560*-containing primed plasmid (pPF189; 6735 bp) and the location of protospacers targeted by new spacers. The map is centered on the location of the primed protospacer, which is indicated with a purple triangle and is on the – strand. Green triangles represent protospacers on the + strand and red triangles show protospacers on the – strand. The histogram (left) indicates the total protospacers on each strand. (**B**) PAM landscape showing the distribution of PAMs throughout pPF189 relative to the primed protospacer (green, + strand and red, – strand). (**C**) Number of total protospacers (blue bars) and protospacers/PAMs (blue line) relative to the primed protospacer. (**D**) Strand-specific protospacer distribution. Bars show number of protospacers, lines indicate protospacers/PAMs and green and red represent protospacer on the + and – strands, respectively. In the lines in (B–D) PAMs and # protospacers/# PAMs were calculated using a shifting window size of 5%. The dashed lines indicate the primed protospacer.

A characteristic feature of priming in the Type I-E system is that the majority of new spacers target the same strand as the original priming spacer (8,10,11). The 105 new spacers all mapped to the plasmid and represented 85 unique sequences (Figure 4A and Supplementary Table S3). However, the spacer locations within the different CRISPR arrays showed that they represent at least 100 independent incorporation events (i.e. they were not ancestors or siblings). The most common spacer length was 32 nt (89%) although 12/105 (11%) were 33 nt long. Surprisingly, 42% of new spacers were in the same orientation as the C2S6 priming spacer (i.e. targeted the − strand) and 58% targeted the + strand (Figure 4A; bar graph). Therefore, priming in the Type I-F system is strand-independent.

Although the new spacers targeted both strands of the plasmid, the distribution of protospacers was biased. There was an increase in protospacers clustered near the primed protospacer (Figure 4A and C; bars) that cannot be explained by the distribution of 793 GG PAMs over the plasmid (the PAM ‘landscape’) (Figure 4B). For example, the majority of all protospacers were located in close proximity to the primed protospacer and their frequency diminished the further from the primed protospacer (Figure 4A and C; blue bars). The same trend was observed when using a shifting window size and when the data were corrected for PAM occurrence (Figure 4C; blue line). This biased distribution is consistent with a priming model, in which spacers targeting protospacers closer to the primed protospacer are more likely to be acquired than those targeting more distal locations. Another striking feature of the data was the apparent strand-specificity of protospacers relative to the location of the primed protospacer (Figure 4A and C). The presence of PAMs throughout the plasmid, indicates that an uneven PAM landscape cannot account for these strand-specific trends. Specifically, acquisition of new spacers complementary to the non-target strand (+ strand protospacers in this experiment), predominantly occurred close to the primed protospacer, yet toward the 5′ on the + strand (Figure 4D; green). In contrast, incorporation of new spacers targeting the – strand (targeted relative to priming spacer) was more evenly distributed, occurring both 5′ and 3′ of, yet still clustering close to, the primed protospacer (Figure 4D; red). Therefore, the acquisition of new spacers during priming in the Type I-F systems appears to proceed in a 3′-5′ direction relative to the primed protospacer on the non-target strand and more diffusely in the vicinity of the primed protospacer on the target strand.

### Protospacer orientation affects the bias in spacer incorporation

To test whether protospacer orientation influenced Type I-F priming, we developed a new system with the red fluorescent protein encoded by mCherry to visually detect plasmid loss and eliminate the need to screen antibiotic resistance. Plasmids were created with no protospacer (negative control) or with – or + strand PAM-protospacers matching CRISPR1 Spacer 1 (C1S1), but containing a –1 PAM mutation to trigger priming. Cultures of the WT harboring the no protospacer negative control, or plasmids with – or + strand primed protospacer orientations, were passaged without selection for 5 days and plasmid loss scored (Supplementary Figure S3). Over 5 days there was minimal loss of the control plasmid, while loss of the – and + strand primed plasmids reached ∼15% (Supplementary Figure S3B).

**Figure 5.**
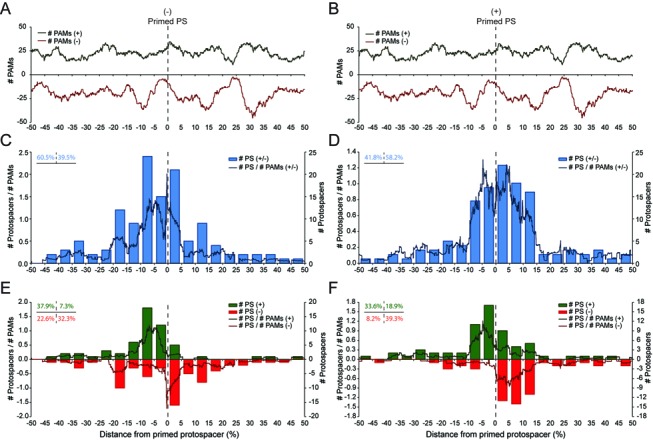
Reversing the direction of the protospacer alters the distribution of spacer incorporation. PAM landscapes of the (**A**) – strand (pPF574; 6034 bp) and (**B**) + strand (pPF575; 6034 bp) primed plasmids. Number of total protospacers and protospacers/PAMs for the (**C**) – strand and (**D**) + strand primed plasmids. Strand-specific protospacer distribution for the (**E**) – strand and (**F**) + strand primed plasmids. Labeling is the same as Figure 4.

The accelerated plasmid loss was due to CRISPR-Cas interference since spacer incorporation into the CRISPR arrays was detected and >120 new spacers for each plasmid were sequenced (Supplementary Tables S4 and S5). The protospacer targets of the new spacers were mapped to both the – and + strand primed plasmids (Supplementary Figure S4A and B), the PAM landscapes (Figure 5A and B), and the overall (Figure 5C and D) and strand-specific (Figure 5E and F) protospacer distributions were determined. Consistent with the *eca0560* experiments, these assays demonstrated that spacer acquisition occurs more frequently near the primed protospacer, irrespective of the direction of the primed protospacer, and decreases further from the primed protospacer (Figure 5C and D). Interestingly, for the – strand primed plasmid, the data paralleled the earlier results with the *eca0560* plasmid (also – strand primed), such that the acquisition of new spacers targeting the original non-target (+) strand, mainly occurred close to, yet 5′ of the primed protospacer (Figure 4D versus Figure 5E; green bars). Again, similar to the data in Figure 4, new spacers targeting the – strand (same target strand as original priming spacer) were more evenly distributed, occurring either side of, yet still close to, the primed protospacer (Figure 5E; red). The opposite trends were detected when the protospacer was reversed (+ strand primed protospacer), with new protospacers on the original non-target (–) strand close to, but 5′ of, the primed protospacer on the – strand (Figure 5F; red) and protospacers on the opposite strand on either side of the primed protospacer (Figure 5F; green). In summary, priming in the Type I-F system leads to efficient acquisition of spacers targeting either strand of the invader. These spacers target close to the original primed protospacer, yet with an uneven strand distribution.

### Priming demonstrates a GG PAM in Type I-F systems

**Figure 6.**
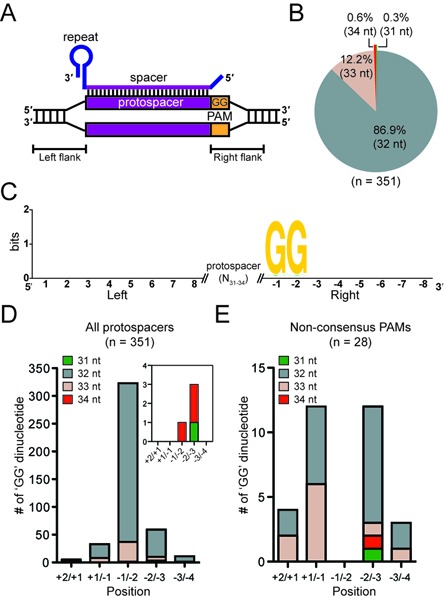
The Type I-F PAM. (**A**) Schematic of the crRNA bound to the protospacer showing the location of the PAM. (**B**) Spacer length distribution. (**C**) Sequence Logo of 8 nt of 5′ and 3′ protospacer flanking sequences. The consensus –1/–2 GG PAM is shown. Number of GG dinucleotides at each position for (**D**) all protospacers and (**E**) protospacers lacking the consensus –1/–2 GG grouped according to spacer length. In (D) the inset shows the GG dinucleotides for the 31 and 34 nt spacers.

The Type I-F system of *P. atrosepticum* contains CRISPR-4 (cluster 4) repeats (34), which were proposed in a bioinformatic study to possess a 5′-protospacer-GG-3′ PAM (21) (Figure 6A). However, no study has analyzed Type I-F PAMs from a large number of experimental acquisition events. Therefore, we combined our data to determine the Type I-F PAM for new spacers incorporated through priming (*n* = 351). Of the new spacers, 87% were 32 nt, 12% were 33 nt, <1% were 34 nt and one case of 31 nt was detected (Figure 6B). The 5′ and 3′ flanks of all protospacers were aligned and sequence logos generated (Figure 6C). No conservation was detected in the 5′ flanking sequence or in the protospacer (Supplementary Figure S5), but a GG PAM was detected in the –1 and –2 positions of the 3′ flanking sequence (i.e. a 5′-protospacer-GG-3′ PAM). When protospacer flanks were analyzed in separate groups based on length, the 32 and 33 nt groups displayed the same overall GG PAM (Figure 6D and Supplementary Figure S5A and B).

Next, we examined protospacers with non-consensus PAMs (*n* = 28). When these were all aligned, GG PAMs were detected at the +1/–1 and –2/–3 positions in 24 of 28 examples (i.e. shifted by ±1 nt relative to the original PAM location) (Figure 6E and Supplementary Figure S5C and D). In *E. coli* these were recently termed ‘slippage’ events (14). The size distribution of these non-PAM spacers was overrepresented for 33 nt (25% compared with 12%) (Figure 6E versus D). When the 33 nt protospacers lacking a consensus –1/–2 PAM (*n* = 7) were analyzed, six contained a +1/–1 GG (Figure 6E and Supplementary Figure S5D). Interestingly, this results in protospacers which are 32 nt from the ‘correct’ –1/–2 PAM location. In conclusion, our data demonstrates that during priming in the I-F system, spacers are preferentially selected that match protospacers with a 5′-protospacer-GG-3′ PAM and infrequently, spacers match protospacers with PAMs shifted by ±1 nt.

## DISCUSSION

Our results have revealed that CRISPR adaptation via priming occurs in the Type I-F CRISPR-Cas system. When pre-existing spacers in the wild-type CRISPRs match protospacers in invading plasmids that lack a consensus PAM, plasmid removal occurs and is accompanied by acquisition of new spacers into all three chromosomal CRISPR arrays. The new spacers interfere with transformation and conjugation and inhibition increases with more spacers. The spacers target both strands of the invader and these protospacers are clustered close to the initial primed protospacer. A strand-independent bias for new spacers occurs that suggests a model of priming that differs from that proposed for the Type I-E system.

Priming in the Type I-E system has only been observed upon artificial overexpression of the *cas* operon, either by deletion of the *hns* repressor (11,15) or through the use of inducible *cas* gene expression (8,10). In contrast, we have shown that the native expression of the Type I-F *cas* genes is sufficient to provoke a priming response with nascent spacers incorporated into all three chromosomal CRISPRs. Remarkably, the number of acquisition events per CRISPR array closely correlated with the original number of existing spacers in these three loci, providing evidence that the length of an array can be an indicator of its incorporation activity. The Type I-F system of *P. atrosepticum* appears the most active characterized system for priming, with up to nine new spacers acquired in a single strain. In the I-E system of *E. coli* a maximum of five or six new spacers has been detected (8,11).

The newly incorporated spacers interfered with the acquisition of plasmids via either transformation or conjugation. Type I-F systems have previously been shown to inhibit growth upon chromosomal self-targeting (32), phage infection (18,35) and plasmid transformation (36), but conjugation has not been examined. Surprisingly, although the new spacers in CRISPR1 and CRISPR2 impeded plasmid uptake, CRISPR3 was inactive for interference, despite acquiring new spacers. All three CRISPR arrays are expressed and processed by Cas6f into ∼60 nt crRNAs; however, CRISPR3 is the most weakly expressed (28). Consistent with the interference deficit of CRISPR3, no PIMs were isolated that contained new spacers only in CRISPR3; any incorporations were accompanied by integrations in CRISPR1 and/or CRISPR2. Whether low expression or sequence deviation within the CRISPR3 repeats account for the lack of targeting requires further investigation. For CRISPR1 and CRISPR2, the level of protection against conjugation was dependent on the number of spacers, with increased protection with up to nine new spacers, independent of whether the leading or lagging strand was targeted (37). This is consistent with the proposal that priming allows not only a rapid response to ‘escape’ mutants, but that by acquiring multiple spacers the response is strengthened and would reduce the possibility of further escape (11).

By analyzing ∼350 newly acquired spacers, we have been able to experimentally define the PAM used in priming adaptation in the Type I-F system (5′-protospacer-GG-3′). This is identical to the PAM predicted computationally by Mojica *et al.* (21) and matches the PAM observed in a few spacers incorporated in *P. aeruginosa* (18). This GG PAM is consistent with those shown previously to be involved in productive interference in the Type I-F systems (18,21,32,36). Interestingly, less than 10% of new spacers had targets that lacked a GG at the consensus –1/–2 position. Most of these contained a GG PAM shifted by ±1 nt, such that GG was at either the +1/–1 or the –2/–3 position. Whether the +1/–1 PAMs enable interference is not known, whereas the –2/–3 PAM ‘slippage’ events result in a G at position –3, which is sufficient for interference in an *E. coli* Type I-F system (36). This suggests that the Type I-F system can make ‘errors’ during spacer selection relative to the PAM location; however, some of these spacers may still be functional for interference.

**Figure 7.**
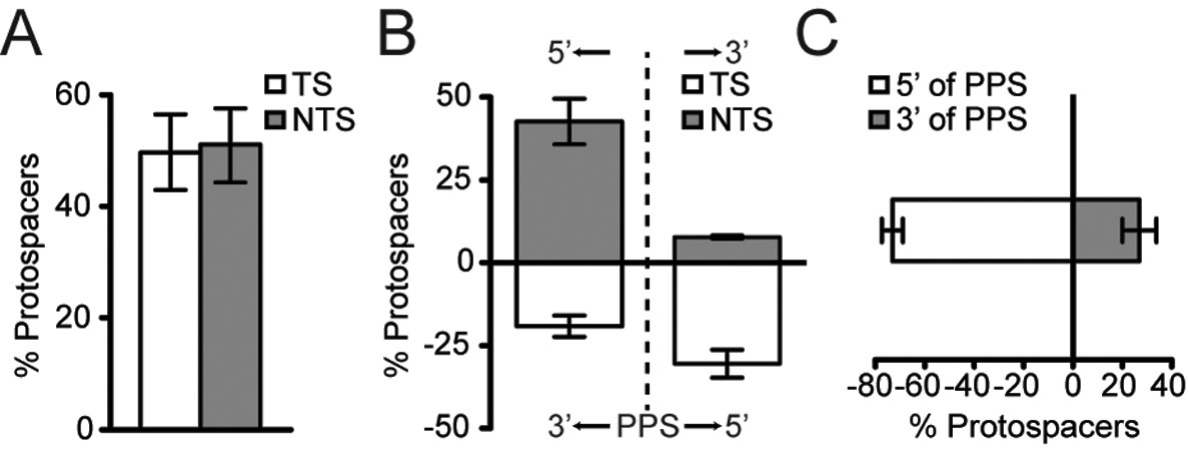
Summary of the protospacer bias during priming. (A) Percentage of protospacers on the target strand (TS) versus the non-target strand (NTS). (B) Percentage of protospacers on the TS and the NTS in the 5′ and 3′ directions relative to the primed protospacer (PPS; dashed line). (C) Protospacer distribution 5′ and 3′ of the PPS (i.e. in opposite directions on each strand relative to the PPS). Data shown are the mean ± SD for the three different plasmids.

Priming in the Type I-F system results in the incorporation of new spacers that target either strand of the invader. Indeed, across three independent experiments there was an equal number of new spacers targeting either DNA strand (Figure 7A). Despite this equal strand distribution, there is a bias in the target locations of the newly acquired spacers, with an obvious enrichment for protospacers close to the location of the initial primed protospacer. Furthermore, the strand of the primed protospacer influences the location and strand targeted by new spacers. Specifically, the priming spacer is complementary to the primed protospacer (the target strand) and new protospacers on the non-target strand (relative to original priming event) are preferentially located 5′ (∼43%) compared with 3′ (<8%) of the primed protospacer (Figure 7B). On the target strand, new protospacers are also more commonly located 5′ of the primed protospacer (∼30%) compared with 3′ (∼20%), but this distribution is more even than the non-target strand bias (Figure 7B). Overall, on both the target and non-target strands there is a strong protospacer location bias with ∼75% 5′ and only ∼25% 3′ relative to the original, primed protospacer (Figure 7C).

**Figure 8.**
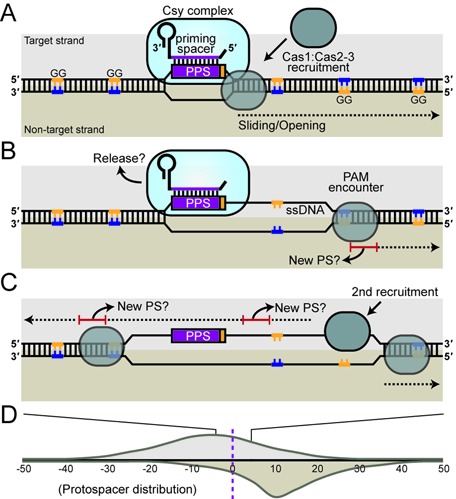
Proposed model of primed acquisition in Type I-F CRISPR-Cas systems. (A) The Csy:crRNA complex binds the target DNA strand at the complementary primed protospacer (PPS) lacking a consensus PAM and generates an R-loop, which displaces the non-target strand. An adaptation complex of Cas1:Cas2–3 is recruited and initiates translocation along the DNA in a 3′-5′ direction on the non-target strand. (B) The Csy:crRNA complex might be released as Cas1:Cas2–3 translocates until it recognizes a PAM (not necessarily the first PAM). New spacers are incorporated that preferentially match protospacers (PS) on the non-target strand. (C) Cas1:Cas2–3 is then predicted to bind to the ssDNA region on the target strand, exposed due to the helicase activity of Cas2–3, and translocate in a 3′-5′ direction on the target strand generating new spacers from suitable PAM locations that match protospacers on the target strand. (D) This bi-directionality results in skewed protospacer distributions, as depicted here schematically.

Based on our data and previous studies we propose a working model for priming in the Type I-F CRISPR-Cas systems (Figure 8). First, a Csy complex forms that contains a crRNA with the priming spacer that guides the complex to the invader dsDNA (26,29). The lack of a consensus PAM (and possibly other protospacer mismatches (15)) is likely to result in weak/infrequent binding of the Csy:crRNA complex to the protospacer target strand in the invader and causes the formation of an R loop with the non-target strand displaced (38). After R loop formation, the native Cas2–3 hybrid protein, in an adaptation complex with Cas1 (26), is recruited to the displaced ssDNA non-target strand, potentially via Csy1 (26,39–41). Cas3 proteins possess adenosine triphosphate-dependent helicase activity and unwind dsDNA in a 3′-5′ direction, while the HD nuclease domain cuts the translocating strand (41–43). We propose that the interaction between Cas1 and Cas2–3 results in a translocating adaptation complex that, upon encountering a GG PAM, generates a new spacer and integrates this into one of the CRISPR arrays. This could account for the preferential location of protospacers 5′ of the primed protospacer on the non-target strand. Whether this new spacer is derived from dsDNA or from one or the other strand is not known. We hypothesize that the helicase activity and movement of the Cas1:Cas2–3 complex along the non-target strand in the 3′-5′ direction results in the displacement of the target strand, and this ssDNA might aid the recruitment of Cas1:Cas2–3 complexes that will translocate in the 3′-5′ direction on the target strand (41). Since the target strand begins to be exposed as ssDNA 3′ of the primed protospacer, the acquisition of new spacers that target this strand initiates 3′ of the primed protospacer and proceeds in the 3′-5′ direction. This would result in the target strand protospacer distribution we observe (Figures 7B and 8D), where protospacers are present on either side of the primed location, but still with a preference towards the 5′ on the target strand. The molecular details of this model will require testing in future studies.

This model for priming in the Type I-F CRISPR-Cas system is in contrast to the I-E system in *E. coli*, where priming results in the preferential acquisition of new spacers that target the same strand as the original priming spacer (8,11). In addition, protospacer clustering is not observed in the Type I-E system, where there was no apparent trend in the target location of new spacers during priming when small plasmids with a limited number of PAMs were analyzed (10). Therefore, despite being evolutionary related (7), the mechanism of priming in the Type I-F system is clearly different from the Type I-E system. A recent study showed that priming occurs in the Type I-B system of *H. hispanica* (20,25). Spacers were acquired that target either strand, but there was an apparent strand bias on either side of the primed protospacer. It was proposed that following Cascade binding and R-loop formation, Cas3 is recruited to the displaced non-target strand and nicks it. Next, in a possible Cascade replacement process, Cas3 was hypothesized to bind the target strand near the primed protospacer. The movement of Cas3 along the ssDNA in a 3′-5′ direction on both strands from the primed protospacer might account for the biased distribution of new protospacers. The adaptation data for the Type I-F system is therefore more similar to the Type I-B than to the I-E system. The higher resolution Type I-F data and our protospacer reversal experiments revealed spacer acquisition trends that have led us to a related, yet distinct model to the one proposed by Li *et al.* (20). Crucially, we provide data that the frequency of spacer acquisition decreases with increasing distance from the primed protospacer, which supports a Cas1:Cas2–3 translocation model where priming proximal PAMs are more likely to be substrates for new spacers (Figure 8). Moreover, the different spacer distributions that we detect on the target versus non-target strands suggest that the recruitment of Cas1:Cas2–3 to the target strand occurs at multiple positions following displacement of the target strand as Cas2–3 translocates along the non-target strand. Further work is required to determine if the Type I-B and I-F systems use the same mechanism for priming, or if they are subtly different. The unique Cas2–3 fusion in the Type I-F systems might indicate that priming proceeds in a different manner. In summary, the general phenomenon of priming adaptation is conserved between CRISPR-Cas subtypes but significant differences exist. The exact molecular mechanisms underlying these processes now need to be addressed.

## SUPPLEMENTARY DATA

Supplementary data are available at NAR Online.

SUPPLEMENTARY DATA
