# Supplementary material for: Priming in the Type I-F CRISPR-Cas system triggers strand-independent spacer acquisition, bi-directionally from the primed protospacer
Source: Nucleic Acids Res. 2014 Jul 1;42(13):8516–26. doi: 10.1093/nar/gku527 (PMC4117759; doi:10.1093/nar/gku527)
Supplement: SUPPLEMENTARY DATA [file supp_42_13_8516__index.html]

Priming in the Type I-F CRISPR-Cas system triggers strand-independent spacer acquisition, bi-directionally from the primed protospacer — SUPPLEMENTARY DATA 

# Priming in the Type I-F CRISPR-Cas system triggers strand-independent spacer acquisition, bi-directionally from the primed protospacer

## SUPPLEMENTARY DATA

**Files in this Data Supplement:**

- Supplementary Data
