# Supplementary material for: Priming in the Type I-F CRISPR-Cas system triggers strand-independent spacer acquisition, bi-directionally from the primed protospacer
Source: Nucleic Acids Res. 2014 Jul 1;42(13):8516–26. doi: 10.1093/nar/gku527 (PMC4117759; doi:10.1093/nar/gku527)
Supplement: SUPPLEMENTARY DATA [file supp_gku527_nar-01187-h-2014-File010.docx]

**Supplementary Information**

**Priming in the Type I-F CRISPR-Cas system triggers strand-independent spacer acquisition, bi-directionally from the primed protospacer**

Corinna Richter^†^, Ron L. Dy, Rebecca E. McKenzie, Bridget N. J. Watson, Corinda Taylor, James T. Chang, Matthew B. McNeil^†^, Raymond H.J. Staals and Peter C. Fineran*

Department of Microbiology and Immunology, University of Otago, PO Box 56, Dunedin 9054, New Zealand.

*To whom correspondence should be addressed. Tel: +64 (0)3 479 7735; Fax: +64 (0)3 479 8540; E-mail: [peter.fineran@otago.ac.nz](mailto:peter.fineran@otago.ac.nz);.

The authors wish it to be known that, in their opinion, the first three authors should be regarded as joint First Authors

^†^Present addresses: [Corinna Richter], Medical Microbiology*,* Department of Laboratory Medicine Malmö, Lund University, Jan Waldenströms gata 59, SE20502 Malmö, Sweden. [Matthew B. McNeil], Centre de Génétique Moléculaire du CNRS, Gif-sur-Yvette 91198, France.

**SUPPLEMENTARY MATERIALS AND METHODS**

**Construction of plasmids**

Vectors encoding the *P. atrosepticum* Eca0560 protein were constructed as follows. The *eca0560* gene was amplified using primers PF448 and PF449. The product was cloned into the BamHI and SalI sites of pTRB30 (a Km^R^ derivative of pQE-80L), generating plasmid pPF189. To construct a mobilizable derivative, the RP4 *oriT* was amplified from pUTmini-Tn5*lacZ*1 with PF985 and PF986, and cloned into the SphI site of pTRB30, yielding plasmid pPF260. Next, *eca0560* was cut from pPF189 on BamHI and SalI sites and cloned into pPF260, resulting in plasmid pPF142. To construct plasmids containing the target of the PIM23 new CRISPR3 spacer, the protospacer was amplified from pPF189 using primers PF1384 and PF1386. The product was cloned into the SphI and HindIII sites of pTRB30 or pPF260, resulting in plasmids pPF511 and pPF641, respectively.

The gene for expression of mCherry, was amplified by PCR, using pmCherry-N1 as template and primers SBP61 and SBP57. The product was digested with EcoRI and cloned into the EcoRI and SmaI sites of pQE-80LoriT. pQE-80LoriT is a derivative of pQE-80L containing the RP4 *oriT* for conjugal transfer. Plasmids pPF571, pPF574 and pPF575 were constructed by amplifying the tetracycline cassette from pTRB31 using primers PF210 and PF1364 (pPF571), PF1367 (pPF574) or PF1368 (pPF575). PF1367 and PF1368 contain F and R protospacer 1 of *P. atrosepticum* CRISPR1 respectively, with 5′-protospacer-TG-3′ PAMs. The resulting products were cut with NcoI and XhoI and cloned into pQE-80LoriT-mCherry using the BspHI and XhoI sites.

**Generation of ΔHAI2 and ΔHAI2Δ*cas***

Strain ΔHAI2 with pTraG1-16 ([1](#_ENREF_1)) was cured of pTraG1-16 by growing in LB without antibiotic selection at 25°C and subsequent plating on LBA. Colonies were checked for plasmid loss by PCR and the resulting strain ΔHAI2 was confirmed by genome sequencing (unpublished data). Strain ΔHAI2Δ*cas* was constructed by generalized transduction of ΔHAI2 using phage ΦTE ([2](#_ENREF_2)) that had been grown on PCF80 (Δ*cas*::*cat*). Transduction of the Δ*cas::cat* mutation was confirmed by selection for Cm^R^ and PCR.

**Table S1**. Bacterial strains and plasmids used in this study.

| **Strain/Plasmid/Phage** | **Genotype/Phenotype** | **Reference** |
| --- | --- | --- |
| ***Escherichia coli*** |  |  |
| DH5α | F^-^, φ80Δd*lacZM15*, Δ(*lacZYA*–*argF*)U169, *endA1*, *recA1*, *hsdR17* (r_K_^-^m_K_^+^), *deoR*, *thi-1*, *supE44*, λ^-^, *gyrA96*, *relA1* | Gibco/BRL |
| S17-1 λ*pir* | *recA, pro, hsdR, recA::*RP4-2-Tc::Mu, λ*pir,* Tmp^R^, Sp^R^, Sm^R^ | ([3](#_ENREF_3)) |
| ***Pectobacterium atrosepticum*** | | |
| ΔHAI2 | ΔHAI2 (markerless deletion) | This study |
| ΔHAI2Δ*cas* | ΔHAI2-derivative, *cas13csy1234*::*cat* (operon deletion), Cm^R^ | This study |
| PCF80 | Δ*cas13csy1234*::*cat* (operon deletion), Cm^R^ | ([4](#_ENREF_4)) |
| PIMs (01-40) | ΔHAI2 derivatives with new spacers against pPF142 (Table S1) | This study |
| PIMs (41-140) | WT derivatives with new spacers targeting pPF574/pPF575 (Tables S2 & S3) | This study |
| SCRI1043 | Wild type (WT) | ([5](#_ENREF_5)) |
|  |  |  |
| **Plasmids** | | |
| pPF142 | pPF260-derivative containing *eca0560*, Km^R^ | This study |
| pPF189 | pTRB30-derivative containing *eca0560*, Km^R^ | This study |
| pPF260 | pTRB30-derivative with RP4 *oriT*, Km^R^ | This study |
| pPF511 | pTRB30-derivative containing PIM23 CRISPR3 spacer target, Km^R^ | This study |
| pPF571 | pQE-80LoriT-mCherry-derivative, Tc^R^ | This study |
| pPF574 | pQE-80LoriT-mCherry-derivative, CRISPR1spacer 1 F priming plasmid, Tc^R^ | This study |
| pPF575 | pQE-80LoriT-mCherry-derivative, CRISPR1spacer 1 R priming plasmid, Tc^R^ | This study |
| pPF641 | pPF260-derivative containing PIM23 CRISPR3 spacer target, Km^R^ | This study |
| pTraG1-16 | CRISPR1 with 16 bp leader and *eca0560* spacer 1, Ap^R^ | ([1](#_ENREF_1)) |
| pTRB30 | pQE-80L (Qiagen) based expression vector, Ap^R^ replaced by Km^R^, Km^R^ | ([4](#_ENREF_4)) |
| pTRB31 | pQE-80L-derivative, Tc^R^ | Tim Blower; unpublished |
| pQE-80LoriT-mCherry | pQE-80L-derivative containing mCherry, RP4 *oriT*, Ap^R^ | Josh Ramsay; unpublished |
| pUTmini-Tn*5lacZ*1 | Vector for the source of RP4 *oriT*, Ap^R^, Km^R^ | ([3](#_ENREF_3)) |
|  |  |  |

**Table S2**. Oligonucleotides used in this study.

| **Name** | **Sequence (5'-3')** | **Notes** | **Restriction site(s) (underlined)** |
| --- | --- | --- | --- |
|  | | | |
| PF138 | CACACTTTGCTATGCCATAG | F for pBAD30 MCS |  |
| PF139 | GCTACTGCCGCCAGG | R for pBAD30 MCS |  |
| PF174 | CGTTAGAGTGATCGGGCTAC | F for CRISPR1 leader |  |
| PF175 | CAATGGCTCAGGGGATTC | R for CRISPR1 spacer 2 |  |
| PF176 | GGTAACTACCGTAAAATAGGAACG | F for CRISPR2 leader |  |
| PF177 | GCCTTTAAGCGCATGTCG | R for CRISPR2 spacer 2 |  |
| PF178 | CTTTAATAATCTGGTTGTTAGTGTG | F for CRISPR3 leader |  |
| PF179 | CCTCAGAAAGCCGACTTC | R for CRISPR3 spacer 2 |  |
| PF209 | TCGTCTTCACCTCGAGAAATC | F for pQE-80L (and derivatives) MCS |  |
| PF210 | GTCATTACTGGATCTATCAACAGG | R for pQE-80L (and derivatives) MCS |  |
| PF217 | CGACGTAAAACGACGGCCAGT | F for pQE-80L (and derivatives) MCS |  |
| PF218 | GGAAACAGCTATGACCATG | R for pQE-80L (and derivatives) MCS |  |
| PF448 | AGGTGGATCCATGAGCGATCGTTATGTCA | F for *eca0560* | BamHI |
| PF449 | AGGTGTCGACCTACTCACCTCCCACTTGC | R for *eca0560* | SalI |
| PF985 | TTTGCATGCCGGCCAGCCTCGCAG | F for RP4 *oriT* | SphI |
| PF986 | TTTGCATGCAGCGCTTTTCCGCTGC | R for RP4 *oriT* | SphI |
| PF1364 | TTTTCCATGGGTCTGACGCTCAGTGGAACG | F pQE-80L no protospacer | NcoI |
| PF1367 | TTTTCCATGG***ACA****GCAGAGACTATCGATACGGTCTGGACGGATGC*GTCTGACGCTCAGTGGAACG | F pQE-80L *C1S1 protospacer*, ***PAM mutant***, F strand - primed | NcoI |
| PF1368 | TTTTCCATGG*GCATCCGTCCAGACCGTATCGATAGTCTCTGC****TGT***GTCTGACGCTCAGTGGAACG | F pQE-80L *C1S1 protospacer*, ***PAM mutant***, R strand - primed | NcoI |
| PF1384 | GCGCATGCGGCGGTTGTTTATCGGTAAGGG | F protospacer 060 in *eca0560* | SphI |
| PF1386 | CGAAGCTTCCCAGATGGAAGATGTAGAGCG | R protospacer 060 in *eca0560* | HindIII |
| SBP61 | TTTTGAATTCTAAATAAAATAAAGGAGGAGTCCCTTATGGTGAGCAAGGGCGAGGAGGA | F mCherry | EcoRI |
| SBP57 | TTTTGGTCTCTTACTTGTACAGCTCGTCCATGC | R mCherry |  |

**Table S3.** pPF189 (primed *eca0560*) PIMs derived from ΔHAI2

| **PIM** | **CRISPR locus** | **spacer position** | **repeat (5'-3')-28bp** | **spacer #** | **spacer (5'-3')** | **spacer (nt)** | **target nt** | **target** | **-/+^a^** | **PAM^b^** |
| --- | --- | --- | --- | --- | --- | --- | --- | --- | --- | --- |
| **WT** | **2** | **6** | **ATTCACTGCCGTATAGGCAGCTTAGAAA** | **C2S6** | **TCCAGTACTCAGGATCGTGTTGGTACGATAAA** | **32** | **1972-2003** | **eca0560** | **-** | **TGTAAAAT** |
| PIM01 | 1 | -4 | GTTCACTGCCGTACAGGCAGCTTAGAAA | S001 | ATTACCGCCCGTCGGTGGAAAGCCGGTACTAC | 32 | 0624-0655 | eca0560 | - | GGGCGCAC |
| PIM01 | 1 | -3 | GTTCACTGCCGTACAGGCAGCTTAGAAA | S002 | TGCACACAGACAAATGCTGCTGTAGCCGAAAC | 32 | 0214-0245 | eca0560 | + | GGCACCAT |
| PIM01 | 1 | -2 | GTTCACTGCCGTACAGGCAGCTTAGAAA | S003 | CATCGTATTGGGTACGACTCGTGTTGGAAAAA | 32 | 0717-0748 | eca0560 | - | GTATGACC |
| PIM01 | 1 | -1 | GTTCACTGCCGTACAGGCAGCTTAGAAA | S004 | ACTGGCCGATCGCCCCGGTCATACCATCGTAT | 32 | 0693-0724 | eca0560 | - | GGCATAGT |
| PIM01 | 2 | -2 | GTTCACTGCCGTACAGGCAGCTTAGAAA | S005 | ATGATGTTACAGATGAGATGGTCAGACTAAAC | 32 | 6316-6347 | kan | + | TGGCAACG |
| PIM01 | 2 | -1 | GTTCACTGCCGTACAGGCAGCTTAGAAA | S006 | GGCCGTCACATAACTGACTGAAGGGGCCAACG | 32 | 0263-0294 | eca0560 | + | GGTGGGTT |
| PIM01 | 3 | -1 | GTTCACTGCCGTGTAGGTAGCTTAGAAA | S007 | TATTTCCCTAAAGGGTTTATTGAGAATATGTT | 32 | 2817-2848 | bb | - | GGCCAGGT |
| PIM02 | 1 | -1 | GTTCACTGCCGTACAGGCAGCTTAGAAA | S008 | ATGAGCAAACTGAAACGTTTTCATCGCTCTGG | 32 | 2707-2738 | bb | - | GGAAAACG |
| PIM02 | 2 | -1 | GTTCACTGCCGTACAGGCAGCTTAGAAA | S009 | TATAACCAGACCGTTCAGCTGGATATTACGGC | 32 | 2517-2548 | bb | - | GGTACATT |
| PIM03 | 1 | -1 | GTTCACTGCCGTACAGGCAGCTTAGAAA | S010 | AGATAGTCCGGCGACAACAGGGACGCCGTTTT | 32 | 1414-1445 | eca0560 | + | GGATATGG |
| PIM04 | 1 | -3 | GTTCACTGCCGTACAGGCAGCTTAGAAA | S011 | TCGCCCGGCACGGTGCGCTTCAGCCCAGACTC | 32 | 0842-0873 | eca0560 | + | GGTGATTC |
| PIM04 | 1 | -2 | GTTCACTGCCGTACAGGCAGCTTAGAAA | S012 | GTTGGGTGTGTTTCTGCGTCCACATAAATCCC | 32 | 0438-0469 | eca0560 | + | GGCTGCGT |
| PIM04 | 1 | -1 | GTTCACTGCCGTACAGGCAGCTTAGAAA | S013 | AAAACACGGTTGGCCGAACTGTTTATTACCCA | 32 | 0745-0776 | eca0560 | - | TCCAACAC |
| PIM05 | 1 | -1 | GTTCACTGCCGTACAGGCAGCTTAGAAA | S014 | ATCCAAAATCGGATCATAGAGTTTCTTACCGG | 32 | 1292-1323 | eca0560 | + | GGCTTGCG |
| PIM06 | 1 | -1 | GTTCACTGCCGTACAGGCAGCTTAGAAA | S015 | GGCACGGTGCGCTTCAGCCCAGACTCGACGTA | 32 | 0836-0867 | eca0560 | + | GGGCGAGG |
| PIM07 | 1 | -1 | GTTCACTGCCGTACAGGCAGCTTAGAAA | S016 | AAATGCCAACGCTATTAGGCTGGCCCTGCATA | 32 | 1230-1261 | eca0560 | + | GGGCAATA |
| PIM09 | 1 | -2 | GTTCACTGCCGTACAGGCAGCTTAGAAA | S017 | GTTGGGTGTGTTTCTGCGTCCACATAAATCCC | 32 | 0438-0469 | eca0560 | + | GGCTGCGT |
| PIM09 | 1 | -1 | GTTCACTGCCGTACAGGCAGCTTAGAAA | S018 | TCAAGCTAGAGAGTCATTACCCCAGGCGTTTA | 32 | 3108-3139 | bb/rrnB | + | GGCATCAA |
| PIM10 | 2 | -2 | GTTCACTGCCGTACAGGCAGCTTAGAAA | S019 | AATAATGGTAATAAAGAGGCAACAATTTTGTC | 32 | 1363-1394 | eca0560 | + | GGAGAAAC |
| PIM10 | 2 | -1 | GTTCACTGCCGTACAGGCAGCTTAGAAA | S020 | AGACCGATATACACAACACCTCGTTTGCGAAC | 32 | 1489-1520 | eca0560 | + | GGACGCCC |
| PIM11 | 1 | -2 | GTTCACTGCCGTACAGGCAGCTTAGAAA | S021 | ATCTGGATTTGTTCAGAACGCTCGGTTGCCGC | 32 | 2332-2353 | bb | - | GGAGTTCT |
| PIM11 | 1 | -1 | GTTCACTGCCGTACAGGCAGCTTAGAAA | S022 | ATGTTTATAAATATGGCCAGCCACGCTAACCA | 32 | 1580-1611 | eca0560 | + | GGTATTAA |
| PIM11 | 2 | -2 | GTTCACTGCCGTACAGGCAGCTTAGAAA | S023 | AGTGAGGGATGGTGGAGCGGGCGTTCACTTAC | 32 | 2185-2216 | eca0560 | - | GGAATGAT |
| PIM11 | 2 | -1 | GTTCACTGCCGTACAGGCAGCTTAGAAA | S024 | TGCAGCCAAGCTTAATTAGCTGAGCTTGGACT | 32 | 2255-2286 | bb | - | GGTCGACC |
| PIM12 | 2 | -2 | GTTCACTGCCGTACAGGCAGCTTAGAAA | S025 | AGTGAGGGATGGTGGAGCGGGCGTTCACTTAC | 32 | 2185-2216 | eca0560 | - | GGAATGAT |
| PIM12 | 2 | -1 | GTTCACTGCCGTACAGGCAGCTTAGAAA | S026 | CTCTAGATTACGTGCAGTCGATGATAAGCTGT | 32 | 3237-3268 | bb | - | GGCGGATT |
| PIM13 | 1 | -3 | GTTCACTGCCGTACAGGCAGCTTAGAAA | S027 | AGAGCACTGGTTGCATTGGGTGAGCGGCCTGA | 32 | 1051-1082 | eca0560 | - | GGCTACAA |
| PIM13 | 1 | -2 | GTTCACTGCCGTACAGGCAGCTTAGAAA | S028 | GATCTGGTTAGCGTGGCTGGCCATATTTATAA | 32 | 1576-1607 | eca0560 | - | GGCAAACA |
| PIM13 | 1 | -1 | GTTCACTGCCGTACAGGCAGCTTAGAAA | S029 | TCGCCCGGCACGGTGCGCTTCAGCCCAGACTC | 32 | 0842-0873 | eca0560 | + | GGTGATTC |
| PIM14 | 1 | -1 | GTTCACTGCCGTACAGGCAGCTTAGAAA | S030 | CAAATGCCAACGCTATTAGGCTGGCCCTGCAT | 32 | 1231-1262 | eca0560 | + | GGCAATAG |
| PIM16 | 1 | -4 | GTTCACTGCCGTACAGGCAGCTTAGAAA | S031 | TAAGTTATTGCGTATCTGCAGTTGTAGCTCGG | 32 | 1166-1197 | eca0560 | + | GGGGTATT |
| PIM16 | 1 | -3 | GTTCACTGCCGTACAGGCAGCTTAGAAA | S032 | TAATAGCGTTGGCATTTGGGCAATAGAGGTTG | 32 | 1245-1276 | eca0560 | - | GGCTGGCC |
| PIM16 | 1 | -2 | GTTCACTGCCGTACAGGCAGCTTAGAAA | S033 | CCAGAGATACGCGACGCCACTTCAGAGACACGC | 33 | 0948-0980 | eca0560 | + | GCAATTGG |
| PIM16 | 1 | -1 | GTTCACTGCCGTACAGGCAGCTTAGAAA | S034 | TGCATTGCGATGAGTTCAATGAGCTAATGGGT | 32 | 1664-1695 | eca0560 | - | GGTTAATG |
| PIM16 | 2 | -1 | GTTCACTGCCGTACAGGCAGCTTAGAAA | S035 | ACGCAAACTCTTTAAATGCAGCACTGTTCCCT | 32 | 0996-1027 | eca0560 | + | GGCGTTTC |
| PIM17 | 2 | -1 | GTTCACTGCCGTACAGGCAGCTTAGAAA | S036 | AATTGCCCAGAGATACGCGACGCCACTTCAGA | 32 | 0955-0986 | eca0560 | + | GGCGGGGG |

^a^+/- refers to the protospacer targeted strand on the plasmid.

^b^PAM is defined as 5’-protospacer-PAM-3’ on the targeted strand (8 nt of flanking sequence is shown).

**Table S3 continued.** pPF189 (primed *eca0560*) PIMs derived from ΔHAI2

| **PIM** | **CRISPR locus** | **spacer position** | **repeat (5'-3')-28bp** | **spacer #** | **spacer (5'-3')** | **spacer (nt)** | **target nt** | **target** | **-/+^a^** | **PAM^b^** |
| --- | --- | --- | --- | --- | --- | --- | --- | --- | --- | --- |
| PIM18 | 1 | -7 | GTTCACTGCCGTACAGGCAGCTTAGAAA | S037 | TATGGAACTGCCTCGGTGAGTTTTCTCCTTCAT | 33 | 5808-5840 | kan | + | GGATGGCA |
| PIM18 | 1 | -6 | GTTCACTGCCGTACAGGCAGCTTAGAAA | S038 | TGATGATGCATGGTTACTCACCACTGCGATCC | 32 | 6231-6262 | kan | + | GGAGTACG |
| PIM18 | 1 | -5 | GTTCACTGCCGTACAGGCAGCTTAGAAA | S039 | CAATGCAACCAGTGCTCTGGCTACAATATTTA | 32 | 1037-1068 | eca0560 | + | GGTGAGCG |
| PIM18 | 1 | -4 | GTTCACTGCCGTACAGGCAGCTTAGAAA | S040 | TCGCCCGGCACGGTGCGCTTCAGCCCAGACTC | 32 | 0842-0873 | eca0560 | + | GGTGATTC |
| PIM18 | 1 | -3 | GTTCACTGCCGTACAGGCAGCTTAGAAA | S041 | AATTGCCCAGAGATACGCGACGCCACTTCAGA | 32 | 0955-0986 | eca0560 | + | GGCGGGGG |
| PIM18 | 1 | -2 | GTTCACTGCCGTACAGGCAGCTTAGAAA | S042 | AGGATCTTGCCATCCTATGGAACTGCCTCGGT | 32 | 5824-5855 | kan | + | GGTATCGG |
| PIM18 | 1 | -1 | GTTCACTGCCGTACAGGCAGCTTAGAAA | S043 | ACAGCAGAAAGAAGAAAAAGCCGTCATTAACC | 32 | 1632-1663 | eca0560 | - | GGCAGTCC |
| PIM18 | 2 | -2 | GTTCACTGCCGTACAGGCAGCTTAGAAA | S044 | CAAACCGTCCAACGGCGTTATAACGAGCTGAT | 32 | 0915-0946 | eca0560 | + | GGCGTGTC |
| PIM18 | 2 | -1 | GTTCACTGCCGTACAGGCAGCTTAGAAA | S045 | TGATACAGGGTTGGCGGCTGAATATAACGCTC | 32 | 0499-0530 | eca0560 | + | GGCCGCCC |
| PIM19 | 1 | -2 | GTTCACTGCCGTACAGGCAGCTTAGAAA | S046 | AATTGCCCAGAGATACGCGACGCCACTTCAGA | 32 | 0955-0986 | eca0560 | + | GGCGGGGG |
| PIM19 | 1 | -1 | GTTCACTGCCGTACAGGCAGCTTAGAAA | S047 | TGCATTGCGATGAGTTCAATGAGCTAATGGGT | 32 | 1664-1695 | eca0560 | - | GGTTAATG |
| PIM20 | 1 | -2 | GTTCACTGCCGTACAGGCAGCTTAGAAA | S048 | AGTGAGGGATGGTGGAGCGGGCGTTCACTTACT | 33 | 2185-2217 | eca0560 | - | GGAATGAT |
| PIM20 | 1 | -1 | GTTCACTGCCGTACAGGCAGCTTAGAAA | S049 | ACAGCAGAAAGAAGAAAAAGCCGTCATTAACC | 32 | 1632-1663 | eca0560 | - | GGCAGTCC |
| PIM20 | 2 | -2 | GTTCACTGCCGTACAGGCAGCTTAGAAA | S050 | TGAGTACTGGATGTAAAATCCTGTCCAGTCTC | 32 | 1951-1982 | eca0560 | + | GGATCGTG |
| PIM20 | 2 | -1 | GTTCACTGCCGTACAGGCAGCTTAGAAA | S051 | ATATCCAGATAGTCCGGCGACAACAGGGACGC | 32 | 1420-1451 | eca0560 | + | GGATGACT |
| PIM21 | 1 | -2 | GTTCACTGCCGTACAGGCAGCTTAGAAA | S052 | ATATCCAGATAGTCCGGCGACAACAGGGACGC | 32 | 1420-1451 | eca0560 | + | GGATGACT |
| PIM21 | 1 | -1 | GTTCACTGCCGTACAGGCAGCTTAGAAA | S053 | AGTGAGGGATGGTGGAGCGGGCGTTCACTTACT | 33 | 2185-2217 | eca0560 | - | GGAATGAT |
| PIM21 | 2 | -1 | GTTCACTGCCGTACAGGCAGCTTAGAAA | S054 | GACCAATGATATTGATGATGCGATGATGCCAG | 32 | 2106-2137 | eca0560 | - | GGCAGCGG |
| PIM22 | 1 | -3 | GTTCACTGCCGTACAGGCAGCTTAGAAA | S055 | AGCAAACTTGCAGGCTGTGGCGACAGAAATGC | 32 | 2136-2167 | eca0560 | - | GGCATCAT |
| PIM22 | 1 | -2 | GTTCACTGCCGTACAGGCAGCTTAGAAA | S056 | CCTGGGGTAATGACTCTCTAGCTTGAGGCATC | 32 | 3114-3145 | bb | - | CGTTTAAG |
| PIM22 | 1 | -1 | GTTCACTGCCGTACAGGCAGCTTAGAAA | S057 | CTTTGGATCGAAGACAATCGTCACATCACCAC | 32 | 0788-0819 | eca0560 | + | GGTGACCC |
| PIM22 | 2 | -1 | GTTCACTGCCGTACAGGCAGCTTAGAAA | S058 | ATGGCCAGAACAGCGAACCCACCGGCCGTCAC | 32 | 0286-0317 | eca0560 | + | GGTACGAA |
| PIM23 | 1 | -1 | GTTCACTGCCGTACAGGCAGCTTAGAAA | S059 | AATTGCCCAGAGATACGCGACGCCACTTCAGA | 32 | 0955-0986 | eca0560 | + | GGCGGGGG |
| PIM23 | 3 | -1 | GTTCACTGCCGTGTAGGTAGCTTAGAAA | S060 | ATGTAGTACCGGCTTTCCACCGACGGGCGGTA | 32 | 0626-0657 | eca0560 | + | GGTATTGA |
| PIM24 | 1 | -2 | GTTCACTGCCGTACAGGCAGCTTAGAAA | S061 | CCTGATTTTCCACAACTGACCACCTTCAAGCA | 32 | 2063-2094 | eca0560 | + | ATGCCGCT |
| PIM24 | 1 | -1 | GTTCACTGCCGTACAGGCAGCTTAGAAA | S062 | TGCATTGCGATGAGTTCAATGAGCTAATGGGT | 32 | 1664-1695 | eca0560 | - | GGTTAATG |
| PIM25 | 1 | -1 | GTTCACTGCCGTACAGGCAGCTTAGAAA | S063 | TGCGGCAGCTGTGTCGTGAGCAATTCTGCAGT | 32 | 1864-1895 | eca0560 | + | GGTTGAGA |
| PIM25 | 2 | -1 | GTTCACTGCCGTACAGGCAGCTTAGAAA | S064 | AGATCGGCAAACATGCTGTTCCCCACTGCCGT | 32 | 1549-1580 | eca0560 | + | GGTTAGCG |
| PIM27 | 1 | -1 | GTTCACTGCCGTACAGGCAGCTTAGAAA | S065 | AGCCACGCTAACCAGATCGGCAAACATGCTGT | 32 | 1562-1593 | eca0560 | + | GGCCATAT |
| PIM28 | 1 | -2 | GTTCACTGCCGTACAGGCAGCTTAGAAA | S066 | GTCAGCCAGTTTAGTCTGACCATCTCATCTGT | 32 | 6308-6339 | kan | - | GGAATTTA |
| PIM28 | 1 | -1 | GTTCACTGCCGTACAGGCAGCTTAGAAA | S067 | TTAACGTGAGTTTTCGTTCCACTGAGCGTCAGA | 33 | 5618-5650 | bb | + | GGGATTTT |
| PIM29 | 1 | -3 | GTTCACTGCCGTACAGGCAGCTTAGAAA | S068 | GTCTGATGTCGTCAGCCTGCCTAAAGGCCAGG | 32 | 2022-2053 | eca0560 | - | GGCTCAAT |
| PIM29 | 1 | -2 | GTTCACTGCCGTACAGGCAGCTTAGAAA | S069 | TCGATATCTGATGAAGTTTGCGTATAGGCGGT | 32 | 1747-1778 | eca0560 | + | GGCGCGTA |
| PIM29 | 1 | -1 | GTTCACTGCCGTACAGGCAGCTTAGAAA | S070 | TGCAGCCAAGCTTAATTAGCTGAGCTTGGACT | 32 | 2255-2286 | bb | - | GGTCGACC |

^a^+/- refers to the protospacer targeted strand on the plasmid.

^b^PAM is defined as 5’-protospacer-PAM-3’ on the targeted strand (8 nt of flanking sequence is shown).

**Table S3 continued.** pPF189 (primed *eca0560*) PIMs derived from ΔHAI2

| **PIM** | **CRISPR locus** | **spacer position** | **repeat (5'-3')-28bp** | **spacer #** | **spacer (5'-3')** | **spacer (nt)** | **target nt** | **target** | **-/+^a^** | **PAM^b^** |
| --- | --- | --- | --- | --- | --- | --- | --- | --- | --- | --- |
| PIM30 | 1 | -5 | GTTCACTGCCGTACAGGCAGCTTAGAAA | S071 | GAACTGAGATACCTACAGCGTGAGCTATGAGA | 32 | 5192-5223 | ori | + | GGTGTAGG |
| PIM30 | 1 | -4 | GTTCACTGCCGTACAGGCAGCTTAGAAA | S072 | GGCCTCTGAGCTTAACGCAACCTCTATTGCCC | 32 | 1262-1293 | eca0560 | + | GGTAAGAA |
| PIM30 | 1 | -3 | GTTCACTGCCGTACAGGCAGCTTAGAAA | S073 | AGACCGTTCAGCTGGATATTACGGCCTTTTTAA | 33 | 2524-2556 | bb | - | GGTTATAG |
| PIM30 | 1 | -2 | GTTCACTGCCGTACAGGCAGCTTAGAAA | S074 | ATGTTTATAAATATGGCCAGCCACGCTAACCAG | 33 | 1579-1611 | eca0560 | + | GGTATTAA |
| PIM30 | 1 | -1 | GTTCACTGCCGTACAGGCAGCTTAGAAA | S075 | ACCGTTGATATATCCCAATGGCATCGTAAAGA | 32 | 2448-2479 | bb | - | GGTATATC |
| PIM30 | 2 | -3 | GTTCACTGCCGTACAGGCAGCTTAGAAA | S076 | TGGGGTAATGACTCTCTAGCTTGAGGCATCAA | 32 | 3116-3147 | bb | - | GGCGTTTA |
| PIM30 | 2 | -2 | GTTCACTGCCGTACAGGCAGCTTAGAAA | S077 | AGATGGAGTTCTGAGGTCATTACTGGATCTAT | 32 | 2294-2325 | bb | + | GGATTTGT |
| PIM30 | 2 | -1 | GTTCACTGCCGTACAGGCAGCTTAGAAA | S078 | TCGCGCGTTTCGGTGATGACGGTGAAAACCTC | 32 | 4540-4571 | bb | - | GGCAGCTC |
| PIM31 | 1 | -3 | GTTCACTGCCGTACAGGCAGCTTAGAAA | S079 | AGTTTTGATTTAAACGTGGCCAATATGGACAA | 32 | 2883-2914 | bb | - | GGTGAAAC |
| PIM31 | 1 | -2 | GTTCACTGCCGTACAGGCAGCTTAGAAA | S080 | CACTGCGATCCCCGGGAAAACAGCATTCCAGG | 32 | 6210-6241 | kan | + | GTGAGTAA |
| PIM31 | 1 | -1 | GTTCACTGCCGTACAGGCAGCTTAGAAA | S081 | TGATGATGCATGGTTACTCACCACTGCGATCC | 32 | 6231-6262 | kan | + | GGAGTACG |
| PIM32 | 1 | -4 | GTTCACTGCCGTACAGGCAGCTTAGAAA | S082 | TAAAGGGTTTATTGAGAATATGTTTTTCGTCT | 32 | 1549-1580 | bb | - | GGGAAATA |
| PIM32 | 1 | -3 | GTTCACTGCCGTACAGGCAGCTTAGAAA | S083 | ATGGTGCCTGCACACAGACAAATGCTGCTGTAG | 33 | 0221-0253 | eca0560 | + | GGGCGATT |
| PIM32 | 1 | -2 | GTTCACTGCCGTACAGGCAGCTTAGAAA | S084 | AGTGAGGGATGGTGGAGCGGGCGTTCACTTAC | 32 | 2185-2216 | eca0560 | - | GGAATGAT |
| PIM32 | 1 | -1 | GTTCACTGCCGTACAGGCAGCTTAGAAA | S085 | TAATAGCGTTGGCATTTGGGCAATAGAGGTTG | 32 | 1245-1276 | eca0560 | - | GGCTGGCC |
| PIM32 | 2 | -1 | GTTCACTGCCGTACAGGCAGCTTAGAAA | S086 | AGTGGCATAGTGACATCAACCTCATCAGGCTC | 32 | 0664-0695 | eca0561 | + | GGCCGATC |
| PIM33 | 2 | -2 | GTTCACTGCCGTACAGGCAGCTTAGAAA | S087 | AGTGAGGGATGGTGGAGCGGGCGTTCACTTAC | 32 | 2185-2216 | eca0560 | - | GGAATGAT |
| PIM33 | 2 | -1 | GTTCACTGCCGTACAGGCAGCTTAGAAA | S088 | TCGTTTGCGAACAATTTGTTCCCAATCAAAAA | 32 | 1469-1500 | eca0560 | + | GGTGTTGT |
| PIM34 | 2 | -1 | GTTCACTGCCGTACAGGCAGCTTAGAAA | S089 | GTATACACTCCGCTATCGCTACGTGACTGGGT | 32 | 4686-4717 | bb | + | TGGCTTAA |
| PIM35 | 2 | -3 | GTTCACTGCCGTACAGGCAGCTTAGAAA | S090 | CCCCGTTCAGCCCGACCGCTGCGCCTTATCCG | 32 | 5266-5297 | ori | - | GGTTCGTG |
| PIM35 | 2 | -2 | GTTCACTGCCGTACAGGCAGCTTAGAAA | S091 | CTGGTTAGCGTGGCTGGCCATATTTATAAACA | 32 | 1579-1610 | eca0560 | - | ATCGGCAA |
| PIM35 | 2 | -1 | GTTCACTGCCGTACAGGCAGCTTAGAAA | S092 | GATACGCGCCTCGATATCTGATGAAGTTTGCG | 32 | 1757-1788 | eca0560 | + | GGGAATGC |
| PIM36 | 1 | -1 | GTTCACTGCCGTACAGGCAGCTTAGAAA | S093 | AGATAGTCCGGCGACAACAGGGACGCCGTTTTA | 33 | 1413-1444 | eca0560 | + | GGATATGG |
| PIM36 | 2 | -1 | GTTCACTGCCGTACAGGCAGCTTAGAAA | S094 | ACCGTTGATATATCCCAATGGCATCGTAAAGA | 32 | 2448-2479 | bb | - | GGTATATC |
| PIM37 | 1 | -2 | GTTCACTGCCGTACAGGCAGCTTAGAAA | S095 | CGCCGCTTTACAGGCTTCGACGCCGCTTCGTT | 32 | 4077-4108 | lacI | - | GTGCACAA |
| PIM37 | 1 | -1 | GTTCACTGCCGTACAGGCAGCTTAGAAA | S096 | AGATGCTCCACGCCCAGTCGCGTACCGTCTTC | 32 | 3886-3917 | lacI | - | GGTCGCAT |
| PIM37 | 2 | -1 | GTTCACTGCCGTACAGGCAGCTTAGAAA | S097 | AGATCGGCAAACATGCTGTTCCCCACTGCCGT | 32 | 1549-1580 | eca0560 | + | GGTTAGCG |
| PIM37 | 3 | -1 | GTTCACTGCCGTGTAGGTAGCTTAGAAA | S098 | ATATTGGCCACGTTTAAATCAAAACTGGTGAA | 32 | 2877-2908 | bb | + | GGACAACT |
| PIM38 | 2 | -1 | GTTCACTGCCGTACAGGCAGCTTAGAAA | S099 | ATGGTGCCTGCACACAGACAAATGCTGCTGTA | 32 | 0222-0253 | eca0560 | + | GGGCGATT |
| PIM39 | 1 | -2 | GTTCACTGCCGTACAGGCAGCTTAGAAA | S100 | CTCGAGCAAGACGTTTCCCGTTGAATATGGCTC | 33 | 6500-6532 | kan | - | GCCGCGAT |
| PIM39 | 1 | -1 | GTTCACTGCCGTACAGGCAGCTTAGAAA | S101 | AGCTGAACGGTCTGGTTATAGGTACATTGAGC | 32 | 2505-2536 | bb | + | GGATATTA |
| PIM39 | 2 | -1 | GTTCACTGCCGTACAGGCAGCTTAGAAA | S102 | TGGGTGAGTTTCACCAGTTTTGATTTAAACGT | 32 | 2868-2899 | bb | - | GGGATTGG |
| PIM40 | 1 | -1 | GTTCACTGCCGTACAGGCAGCTTAGAAA | S103 | GAAGACAGCTCATGTTATATCCCGCCGTTAACC | 33 | 3546-3578 | lacI | + | GGTATCGT |
| PIM40 | 2 | -2 | GTTCACTGCCGTACAGGCAGCTTAGAAA | S104 | TGATGATGCATGGTTACTCACCACTGCGATCC | 32 | 6231-6262 | kan | + | GGAGTACG |
| PIM40 | 2 | -1 | GTTCACTGCCGTACAGGCAGCTTAGAAA | S105 | AGAGTTGTTTCTGAAACATGGCAAAGGTAGCGT | 33 | 6353-6385 | kan | + | GGCGCATC |

^a^+/- refers to the protospacer targeted strand on the plasmid.

^b^PAM is defined as 5’-protospacer-PAM-3’ on the targeted strand (8 nt of flanking sequence is shown).

**Table S4.** pPF574 (- strand primed protospacer) PIMs derived from WT *P. atrosepticum*

| **PIM** | **CRISPR locus** | **spacer position** | **repeat (5'-3')-28bp** | **spacer #** | **spacer (5'-3')** | **spacer (nt)** | **target nt** | **target** | **-/+^a^** | **PAM^b^** |
| --- | --- | --- | --- | --- | --- | --- | --- | --- | --- | --- |
| **WT** | **1** | **1** | **GTTCACTGCCGTACAGGCAGCTTAGAAA** | **C1S1** | **GCAGAGACTATCGATACGGTCTGGACGGATGC** | **32** | **4516 - 4550** |  | **-** | **TGCCCATG** |
| PIM41 | 1 | -3 | GTTCACTGCCGTACAGGCAGCTTAGAAA | S106 | ACGCCCAGTCGCGTACCGTCTTCATGGGAGAAA | 33 | 2490 - 2522 | lacI | - | GGAGCATC |
| PIM41 | 1 | -2 | GTTCACTGCCGTACAGGCAGCTTAGAAA | S107 | AATGCGACCAGATGCTCCACGCCCAGTCGCGT | 32 | 2472 - 2503 | lacI | - | GGGTCACC |
| PIM41 | 1 | -1 | GTTCACTGCCGTACAGGCAGCTTAGAAA | S108 | CTGGCGCCCAATACGCAAACCGCCTCTCCCCG | 32 | 1983 - 2014 | lacI | + | GGTGGTTT |
| PIM42 | 1 | -1 | GTTCACTGCCGTACAGGCAGCTTAGAAA | S109 | TGCCCGGCTGACGCCGTTGGATACACCAAGGA | 32 | 3468 - 3499 | RP4/OriT | - | GGATAGGT |
| PIM43 | 1 | -1 | GTTCACTGCCGTACAGGCAGCTTAGAAA | S110 | TACATACCTCGCTCTGCTAATCCTGTTACCAG | 32 | 4207 - 4238 | pBR322_origin | + | GGCGGTGC |
| PIM44 | 1 | -2 | GTTCACTGCCGTACAGGCAGCTTAGAAA | S111 | TACATACCTCGCTCTGCTAATCCTGTTACCAG | 32 | 4207 - 4238 | pBR322_origin | + | GGCGGTGC |
| PIM44 | 1 | -1 | GTTCACTGCCGTACAGGCAGCTTAGAAA | S112 | GCCGGCTTCCATTCAGGTCGAGGTGGCCCGGC | 32 | 4657 - 4688 | TcR | - | GGCACCTC |
| PIM45 | 1 | -1 | GTTCACTGCCGTACAGGCAGCTTAGAAA | S113 | AAGGAAAGTCTACACGAACCCTTTGGCAAAAT | 32 | 3495 - 3526 | RP4/OriT | - | GGTGTATC |
| PIM46 | 1 | -1 | GTTCACTGCCGTACAGGCAGCTTAGAAA | S114 | GAAGGGAGAAAGGCGGACAGGTATCCGGTAAG | 32 | 3993 - 4024 | pBR322_origin | + | GGGAAGCG |
| PIM47 | 1 | -2 | GTTCACTGCCGTACAGGCAGCTTAGAAA | S115 | ATTACCGAGTCCGGGCTGCGCGTTGGTGCGGA | 32 | 2202 - 2233 | lacI | + | GGCGCGCA |
| PIM47 | 1 | -1 | GTTCACTGCCGTACAGGCAGCTTAGAAA | S116 | GTCGGAGGGGAAGTTGGTGCCGCGCAGCTTCAC | 33 | 0498 - 0530 | mCherry | + | GGCCCCGT |
| PIM48 | 1 | -3 | GTTCACTGCCGTACAGGCAGCTTAGAAA | S117 | GCTACCAGCGGTGGTTTGTTTGCCGGATCAAG | 32 | 4354 - 4385 | pBR322_origin | + | GGTGGTTT |
| PIM48 | 1 | -2 | GTTCACTGCCGTACAGGCAGCTTAGAAA | S118 | TGAATGGAAGCCGGCGGCACCTCGCTAACGGAT | 33 | 4639 - 4671 | TcR | + | GGTCGAGG |
| PIM48 | 1 | -1 | GTTCACTGCCGTACAGGCAGCTTAGAAA | S119 | TGTCCTACGAGTTGCATGATAAAGAAGACAGT | 32 | 5180 - 5211 | TcR | - | GGTGCCGG |
| PIM49 | 1 | -1 | GTTCACTGCCGTACAGGCAGCTTAGAAA | S120 | TACATACCTCGCTCTGCTAATCCTGTTACCAG | 32 | 4207 - 4238 | pBR322_origin | + | GGCGGTGC |
| PIM50 | 1 | -3 | GTTCACTGCCGTACAGGCAGCTTAGAAA | S121 | GGTAAGCGGCAGGGTCGGAACAGGAGAGCGCA | 32 | 3967 - 3998 | pBR322_origin | + | GGATACCT |
| PIM50 | 1 | -2 | GTTCACTGCCGTACAGGCAGCTTAGAAA | S122 | TGGGACATCCTGTCCCCTCAGTTCATGTACGG | 32 | 0306 - 0337 | mCherry | - | GGCGAAGG |
| PIM50 | 1 | -1 | GTTCACTGCCGTACAGGCAGCTTAGAAA | S123 | TCGGCGCGTTCGTACTGTTCCACGATGGTGTA | 32 | 0756 - 0787 | mCherry | + | GGGCCGCC |
| PIM50 | 2 | -1 | GTTCACTGCCGTACAGGCAGCTTAGAAA | S124 | TTTCTCCCTTCGGGAAGCGTGGCGCTTTCTCA | 32 | 4014 - 4045 | pBR322_origin | - | GGCGGACA |
| PIM51 | 1 | -3 | GTTCACTGCCGTACAGGCAGCTTAGAAA | S125 | TACATACCTCGCTCTGCTAATCCTGTTACCAG | 32 | 4207 - 4238 | pBR322_origin | + | GGCGGTGC |
| PIM51 | 1 | -2 | GTTCACTGCCGTACAGGCAGCTTAGAAA | S126 | TTAGCGAGGTGCCGCCGGCTTCCATTCAGGTC | 32 | 4644 - 4675 | TcR | - | CGGATTCA |
| PIM51 | 1 | -1 | GTTCACTGCCGTACAGGCAGCTTAGAAA | S127 | ATTCAGGTCGAGGTGGCCCGGCTCCATGCACC | 32 | 4667 - 4698 | TcR | - | GGAAGCCG |
| PIM51 | 2 | -1 | GTTCACTGCCGTACAGGCAGCTTAGAAA | S128 | GTTGAGCACCGCCAGGTGCGAATAAGGGACAG | 32 | 3391 - 3422 | RP4/OriT | - | GGGAATCC |
| PIM52 | 1 | -1 | GTTCACTGCCGTACAGGCAGCTTAGAAA | S129 | TGAATGGAAGCCGGCGGCACCTCGCTAACGGA | 32 | 4640 - 4671 | TcR | + | GGTCGAGG |
| PIM52 | 1 | -2 | GTTCACTGCCGTACAGGCAGCTTAGAAA | S130 | AACCCGTTCCATGTGCTCGCCGAGGCGGCATAA | 33 | 4754 - 4786 | TcR | - | GGCATGGA |
| PIM52 | 2 | -1 | GTTCACTGCCGTACAGGCAGCTTAGAAA | S131 | GGATCAAGAGCTACCAACTCTTTTTCCGAAGG | 32 | 4330 - 4361 | pBR322_origin | + | GGCAAACA |
| PIM52 | 2 | -2 | GTTCACTGCCGTACAGGCAGCTTAGAAA | S132 | GAAAATGACCCAGAGCGCTGCCGGCACCTGTC | 32 | 5152 - 5183 | TcR | - | GGCGAGGA |
| PIM53 | 1 | -1 | GTTCACTGCCGTACAGGCAGCTTAGAAA | S133 | GTTGGATACACCAAGGAAAGTCTACACGAACC | 32 | 3483 - 3514 | RP4/OriT | - | GGCGTCAG |
| PIM53 | 1 | -2 | GTTCACTGCCGTACAGGCAGCTTAGAAA | S134 | GAACCCGTTCCATGTGCTCGCCGAGGCGGCATAA | 34 | 4753 - 4786 | TcR | - | GCATGGAT |
| PIM53 | 2 | -1 | GTTCACTGCCGTACAGGCAGCTTAGAAA | S135 | ACACAGTCCCCCGGCCACGGGGCCTGCCACCA | 32 | 5399 - 5429 | TcR | - | GGCGCCAT |
| PIM53 | 2 | -2 | GTTCACTGCCGTACAGGCAGCTTAGAAA | S136 | TGAATGGAAGCCGGCGGCACCTCGCTAACGGA | 32 | 4640 - 4671 | TcR | + | GGTCGAGG |
| PIM53 | 2 | -3 | GTTCACTGCCGTACAGGCAGCTTAGAAA | S137 | GCCGACATCCCCGACTACTTGAAGCTGTCCTT | 32 | 0363 - 0394 | mCherry | - | GGGGTGCT |
| PIM53 | 2 | -4 | GTTCACTGCCGTACAGGCAGCTTAGAAA | S138 | TACACCGAACTGAGATACCTACAGCGTGAGCT | 32 | 4047 - 4078 | pBR322_origin | + | GGTCGTTC |
| PIM54 | 1 | -2 | GTTCACTGCCGTACAGGCAGCTTAGAAA | S139 | AGTGGCTGCTGCCAGTGGCGATAAGTCGTGTC | 32 | 4177 - 4208 | pBR322_origin | + | GGTAACAG |
| PIM54 | 1 | -1 | GTTCACTGCCGTACAGGCAGCTTAGAAA | S140 | AACCCGTTCCATGTGCTCGCCGAGGCGGCATA | 32 | 4754 - 4785 | TcR | - | GGCATGGA |
| PIM55 | 1 | -3 | GTTCACTGCCGTACAGGCAGCTTAGAAA | S141 | TTCTAGTGTAGCCGTAGTTAGGCCACCACTTC | 32 | 4259 - 4290 | pBR322_origin | + | GGACAGTA |
| PIM55 | 1 | -2 | GTTCACTGCCGTACAGGCAGCTTAGAAA | S142 | GAAACAAGCGCTCATGAGCCCGAAGTGGCGAG | 32 | 5440 - 5471 | TcR | - | GGCGTGGG |
| PIM55 | 2 | -1 | GTTCACTGCCGTACAGGCAGCTTAGAAA | S143 | GTTAGCGAGGTGCCGCCGGCTTCCATTCAGGT | 32 | 4643 - 4674 | TcR | - | GGATTCAC |
| PIM56 | 1 | -3 | GTTCACTGCCGTACAGGCAGCTTAGAAA | S144 | GTCATCACCGAAACGCGCGAGGCAGCTCTAGAT | 33 | 3122 - 3154 | bb | + | GGTGAAAA |
| PIM56 | 1 | -2 | GTTCACTGCCGTACAGGCAGCTTAGAAA | S145 | CGAAATTCCGACACCATCGAATGGTGCAAAAC | 32 | 3057 - 3088 | bb | + | GGCAGCGT |
| PIM56 | 1 | -1 | GTTCACTGCCGTACAGGCAGCTTAGAAA | S146 | CTTCGGGAAGCGTGGCGCTTTCTCATAGCTCAC | 33 | 4021 - 4053 | pBR322_origin | - | GGAGAAAG |

^a^+/- refers to the protospacer targeted strand on the plasmid.

^b^PAM is defined as 5’-protospacer-PAM-3’ on the targeted strand (8 nt of flanking sequence is shown).

**Table S4 continued.** pPF574 (- strand primed protospacer) PIMs derived from WT *P. atrosepticum*

| **PIM** | **CRISPR locus** | **spacer position** | **repeat (5'-3')-28bp** | **spacer #** | **spacer (5'-3')** | **spacer (nt)** | **target nt** | **target** | **-/+^a^** | **PAM^b^** |
| --- | --- | --- | --- | --- | --- | --- | --- | --- | --- | --- |
| PIM56 | 2 | -1 | GTTCACTGCCGTACAGGCAGCTTAGAAA | S147 | TATTCGCACCTGGCGGTGCTCAACGGGAATCC | 32 | 3383 - 3414 | RP4/OriT | + | AGGGACAG |
| PIM56 | 3 | -1 | GTTCACTGCCGTGTAGGTAGCTTAGAAA | S148 | TTATTCGCACCTGGCGGTGCTCAACGGGAATC | 32 | 3384 - 3415 | RP4/OriT | + | GGGACAGT |
| PIM57 | 1 | -5 | GTTCACTGCCGTACAGGCAGCTTAGAAA | S149 | TTTTGCTGGCCTTTTGCTCACATGTTCTTTCC | 32 | 2221 - 2252 | lacI | + | GGCCAGGA |
| PIM57 | 1 | -4 | GTTCACTGCCGTACAGGCAGCTTAGAAA | S150 | ACCTCCAGTCTGGCCCTGCACGCGCCGTCGCAA | 33 | 2768 - 2800 | lacI | + | GGCAACGC |
| PIM57 | 1 | -3 | GTTCACTGCCGTACAGGCAGCTTAGAAA | S151 | AGGCGTTTCCCCCTGGAAGCTCCCTCGTGCGC | 32 | 3940 - 3971 | pBR322_origin | - | GGTATCTT |
| PIM57 | 1 | -1 | GTTCACTGCCGTACAGGCAGCTTAGAAA | S152 | AGCGGTGGTTTGTTTGCCGGATCAAGAGCTAC | 32 | 4348 - 4379 | pBR322_origin | + | GGTAGCGG |
| PIM58 | 1 | -2 | GTTCACTGCCGTACAGGCAGCTTAGAAA | S153 | GAAGGGAGAAAGGCGGACAGGTATCCGGTAAG | 32 | 3993 - 4024 | pBR322_origin | + | GGGAAGCG |
| PIM58 | 1 | -1 | GTTCACTGCCGTACAGGCAGCTTAGAAA | S154 | ACCGCTACCAGCGGTGGTTTGTTTGCCGGATC | 32 | 4357 - 4388 | pBR322_origin | + | GGTTTTTT |
| PIM59 | 1 | -1 | GTTCACTGCCGTACAGGCAGCTTAGAAA | S155 | TTATCCGGTAACTATCGTCTTGAGTCCAACCC | 32 | 4140 - 4171 | pBR322_origin | - | GGCGCAGC |
| PIM60 | 1 | -1 | GTTCACTGCCGTACAGGCAGCTTAGAAA | S156 | TTCTAGTGTAGCCGTAGTTAGGCCACCACTTC | 32 | 4259 - 4290 | pBR322_origin | + | GGACAGTA |
| PIM61 | 1 | -1 | GTTCACTGCCGTACAGGCAGCTTAGAAA | S157 | CGAAGGGAGAAAGGCGGACAGGTATCCGGTAA | 32 | 3994 - 4025 | pBR322_origin | + | GGAAGCGT |
| PIM62 | 1 | -3 | GTTCACTGCCGTACAGGCAGCTTAGAAA | S158 | ACGCTGGCACCCAGTTGATCGGCGCGAGATTT | 32 | 2721 - 2752 | lacI | - | GGTGGTGT |
| PIM62 | 1 | -2 | GTTCACTGCCGTACAGGCAGCTTAGAAA | S159 | TGGTTCACCACGCGGGAAACGGTCTGATAAGA | 32 | 2925 - 2956 | lacI | - | GGCCAGCC |
| PIM62 | 1 | -1 | GTTCACTGCCGTACAGGCAGCTTAGAAA | S160 | AGACACCCATCAACAGTATTATTTTCTCCCAT | 32 | 2513 - 2544 | lacI | + | GGTCAGAG |
| PIM63 | 1 | -1 | GTTCACTGCCGTACAGGCAGCTTAGAAA | S161 | ACTTCAAGAACTCTGTAGCACCGCCTACATAC | 32 | 4232 - 4263 | pBR322_origin | + | GGTGGCCT |
| PIM64 | 1 | -1 | GTTCACTGCCGTACAGGCAGCTTAGAAA | S162 | CGCTCGCGGGTGGGCCTACTTCACCTATCCTG | 32 | 3438 - 3469 | RP4/OriT | - | GGTGTTCC |
| PIM65 | 1 | -5 | GTTCACTGCCGTACAGGCAGCTTAGAAA | S163 | ATAGTGACTGGCGATGCTGTCGGAATGGACGA | 32 | 5725 - 5756 | TcR | - | GGCGTGCT |
| PIM65 | 1 | -4 | GTTCACTGCCGTACAGGCAGCTTAGAAA | S164 | TCCCACAACGAGGACTACACCATCGTGGAACA | 32 | 741 - 772 | mCherry | - | GGTGATGT |
| PIM65 | 1 | -3 | GTTCACTGCCGTACAGGCAGCTTAGAAA | S165 | ATACCCACGCCGAAACAAGCGCTCATGAGCCC | 32 | 5429 - 5460 | bb | - | GGTGGCAG |
| PIM65 | 1 | -2 | GTTCACTGCCGTACAGGCAGCTTAGAAA | S166 | GCTGGTAGCGGTGGTTTTTTTGTTTGCAAGCA | 32 | 4377 - 4408 | pBR322_origin | - | GGTGGTTT |
| PIM65 | 1 | -1 | GTTCACTGCCGTACAGGCAGCTTAGAAA | S167 | TCGCTCTGCTAATCCTGTTACCAGTGGCTGCT | 32 | 4199 - 4230 | pBR322_origin | + | GGTATGTA |
| PIM66 | 1 | -3 | GTTCACTGCCGTACAGGCAGCTTAGAAA | S168 | TGACTGCGTTAGCAATTTAACTGTGATAAACT | 32 | 5870 - 5901 | bb | - | GGCACCGT |
| PIM66 | 1 | -2 | GTTCACTGCCGTACAGGCAGCTTAGAAA | S169 | CTGATGGTCGTCATCTACCTGCCTGGACAGCAT | 33 | 4861 - 4893 | bb | - | GGACAGCT |
| PIM66 | 1 | -1 | GTTCACTGCCGTACAGGCAGCTTAGAAA | S170 | AGCGGTGGTTTGTTTGCCGGATCAAGAGCTAC | 32 | 4348 - 4379 | pBR322_origin | + | GGTAGCGG |
| PIM67 | 1 | -2 | GTTCACTGCCGTACAGGCAGCTTAGAAA | S171 | AGGATGCCATTGCTGTGGAAGCTGCCTGCACT | 32 | 2579 - 2610 | lacI | + | GGTCATCC |
| PIM67 | 1 | -1 | GTTCACTGCCGTACAGGCAGCTTAGAAA | S172 | TGGAAGCTCCCTCGTGCGCTCTCCTGTTCCGA | 32 | 3953 - 3984 | pBR322_origin | - | GGGGGAAA |
| PIM68 | 1 | -2 | GTTCACTGCCGTACAGGCAGCTTAGAAA | S173 | AGCATCGCAGTGGGAACGATGCCCTCATTCAG | 32 | 2274 - 2305 | lacI | - | GGTTGCCA |
| PIM68 | 1 | -1 | GTTCACTGCCGTACAGGCAGCTTAGAAA | S174 | GTAACACGCCACATCTTGCGAATATATGTGTA | 32 | 1365 - 1396 | bb | + | GGTGAAAA |
| PIM69 | 1 | -3 | GTTCACTGCCGTACAGGCAGCTTAGAAA | S175 | AAAGCGGTCGGACAGTGCTCCGAGAACGGGTG | 32 | 5650 - 5681 | bb | - | GGCCGCCG |
| PIM69 | 1 | -2 | GTTCACTGCCGTACAGGCAGCTTAGAAA | S176 | AAGTAGCGAAGCGAGCAGGACTGGGCGGCGGC | 32 | 5617 - 5648 | bb | - | GGAGCCAC |
| PIM69 | 1 | -1 | GTTCACTGCCGTACAGGCAGCTTAGAAA | S177 | TGTGGCGCCGGTGATGCCGGCCACGATGCGTC | 32 | 5524 - 5555 | bb | - | GGTGCGGT |
| PIM70 | 1 | -3 | GTTCACTGCCGTACAGGCAGCTTAGAAA | S178 | ATGCACCGCGACGCAACGCGGGGAGGCAGACA | 32 | 4692 - 4723 | bb | - | GGAGCCGG |
| PIM70 | 1 | -2 | GTTCACTGCCGTACAGGCAGCTTAGAAA | S179 | TACGAGTTGCATGATAAAGAAGACAGTCATAA | 32 | 5185 - 5216 | bb | - | GGACAGGT |
| PIM70 | 1 | -1 | GTTCACTGCCGTACAGGCAGCTTAGAAA | S180 | GTAGTTAGGCCACCACTTCAAGAACTCTGTAG | 32 | 4246 - 4277 | pBR322_origin | + | GGCTACAC |
| PIM71 | 1 | -1 | GTTCACTGCCGTACAGGCAGCTTAGAAA | S181 | GTTAGCGAGGTGCCGCCGGCTTCCATTCAGGT | 32 | 4643 - 4674 | bb | - | GGATTCAC |
| PIM72 | 1 | -2 | GTTCACTGCCGTACAGGCAGCTTAGAAA | S182 | GTTCAGCCCGACCGCTGCGCCTTATCCGGTAA | 32 | 4119 - 4150 | pBR322_origin | - | GGGGGGTT |
| PIM72 | 1 | -1 | GTTCACTGCCGTACAGGCAGCTTAGAAA | S183 | CGCAGCCGAACGACCGAGCGCAGCGAGTCAGT | 32 | 3687 - 3718 | bb | + | GCGAGCGG |
| PIM73 | 1 | -1 | GTTCACTGCCGTACAGGCAGCTTAGAAA | S184 | AACAGTCCCCCGGCCACGGGGCCTGCCACCAT | 32 | 5399 - 5430 | bb | - | GGGCGCCA |
| PIM74 | 1 | -4 | GTTCACTGCCGTACAGGCAGCTTAGAAA | S185 | GGTGCGAATAAGGGACAGTGAAGAAGGAACA | 31 | 3405 - 3435 | OriT | - | TGGCGGTG |
| PIM74 | 1 | -3 | GTTCACTGCCGTACAGGCAGCTTAGAAA | S186 | TTGAGAGCCTTCAACCCAGTCAGCTCCTTCCG | 32 | 5249 - 5280 | bb | + | GGGCATCG |
| PIM74 | 1 | -2 | GTTCACTGCCGTACAGGCAGCTTAGAAA | S187 | AGCTTGGAGCGAACGACCTACACCGAACTGAGA | 33 | 4064 - 4096 | pBR322_origin | + | GGGCTGTG |
| PIM74 | 1 | -1 | GTTCACTGCCGTACAGGCAGCTTAGAAA | S188 | GCAAGAGGCCCGGCAGTACCGGCATAACCAAG | 32 | 5763 - 5794 | bb | - | GGGATATC |

^a^+/- refers to the protospacer targeted strand on the plasmid.

^b^PAM is defined as 5’-protospacer-PAM-3’ on the targeted strand (8 nt of flanking sequence is shown).

**Table S4 continued.** pPF574 (- strand primed protospacer) PIMs derived from WT *P. atrosepticum*

| **PIM** | **CRISPR locus** | **spacer position** | **repeat (5'-3')-28bp** | **spacer #** | **spacer (5'-3')** | **spacer (nt)** | **target nt** | **target** | **-/+^a^** | **PAM^b^** |
| --- | --- | --- | --- | --- | --- | --- | --- | --- | --- | --- |
| PIM75 | 1 | -4 | GTTCACTGCCGTACAGGCAGCTTAGAAA | S189 | CGCTCGCGGGTGGGCCTACTTCACCTATCCTG | 32 | 3438 - 3469 | OriT | - | GGTGTTCC |
| PIM75 | 1 | -3 | GTTCACTGCCGTACAGGCAGCTTAGAAA | S190 | GCTATCGCTACGTGACTGGGTCATGGCTGCGCC | 33 | 3269 - 3301 | bb | + | GGAGTGTA |
| PIM75 | 1 | -2 | GTTCACTGCCGTACAGGCAGCTTAGAAA | S191 | ACCGCTACCAGCGGTGGTTTGTTTGCCGGATC | 32 | 4357 - 4388 | pBR322_origin | + | GGTTTTTT |
| PIM75 | 1 | -1 | GTTCACTGCCGTACAGGCAGCTTAGAAA | S192 | ATTCAGGTCGAGGTGGCCCGGCTCCATGCACC | 32 | 4667 - 4698 | bb | - | GGAAGCCG |
| PIM76 | 1 | -3 | GTTCACTGCCGTACAGGCAGCTTAGAAA | S193 | TGCGTTATCCCCTGATTCTGTGGATAACCGTA | 32 | 3751 - 3782 | bb | + | GGAAAGAA |
| PIM76 | 1 | -2 | GTTCACTGCCGTACAGGCAGCTTAGAAA | S194 | ACAGAATCAGGGGATAACGCAGGAAAGAACAT | 32 | 3762 - 3793 | bb | - | GGATAACC |
| PIM76 | 1 | -1 | GTTCACTGCCGTACAGGCAGCTTAGAAA | S195 | GCCAGGTGCGAATAAGGGACAGTGAAGAAGGAA | 33 | 3401 - 3433 | OriT | - | GGTGCTCA |
| PIM77 | 1 | -1 | GTTCACTGCCGTACAGGCAGCTTAGAAA | S196 | TGATGGTCGTCATCTACCTGCCTGGACAGCAT | 32 | 4862 - 4893 | bb | - | GGGACAGC |
| PIM78 | 1 | -1 | GTTCACTGCCGTACAGGCAGCTTAGAAA | S197 | TGTCCTACGAGTTGCATGATAAAGAAGACAGTC | 33 | 5180 - 5212 | bb | - | GGTGCCGG |
| PIM79 | 1 | -4 | GTTCACTGCCGTACAGGCAGCTTAGAAA | S198 | TTCGGGAAGCGTGGCGCTTTCTCATAGCTCAC | 32 | 4022 - 4053 | pBR322_origin | - | GGGAGAAA |
| PIM79 | 1 | -3 | GTTCACTGCCGTACAGGCAGCTTAGAAA | S199 | AGTCGGGAAACCTGTCGTGCCAGCTGCATTAA | 32 | 1935 - 1966 | lacI | - | GGAAAGCG |
| PIM79 | 1 | -2 | GTTCACTGCCGTACAGGCAGCTTAGAAA | S200 | TACGAGGGCACCCAGACCGCCAAGCTGAAGGT | 32 | 246 - 277 | mCherry | - | GGGGCGGC |
| PIM79 | 1 | -1 | GTTCACTGCCGTACAGGCAGCTTAGAAA | S201 | TTTTTACGGTTCCTGGCCTTTTGCTGGCCTTT | 32 | 3801 - 3832 | bb | + | GGCCGCGT |
| PIM80 | 1 | -1 | GTTCACTGCCGTACAGGCAGCTTAGAAA | S202 | AGTGGCGATAAGTCGTGTCTTACCGGGTTGGA | 32 | 4164 - 4195 | pBR322_origin | + | GGCAGCAG |
| PIM81 | 1 | -2 | GTTCACTGCCGTACAGGCAGCTTAGAAA | S203 | ATTCAGGTCGAGGTGGCCCGGCTCCATGCACC | 32 | 4667 - 4698 | bb | - | GGAAGCCG |
| PIM81 | 1 | -1 | GTTCACTGCCGTACAGGCAGCTTAGAAA | S204 | TTTTTACGGTTCCTGGCCTTTTGCTGGCCTTT | 32 | 3801 - 3832 | bb | + | GGCCGCGT |
| PIM82 | 1 | -1 | GTTCACTGCCGTACAGGCAGCTTAGAAA | S205 | AGTGGCTGCTGCCAGTGGCGATAAGTCGTGTC | 32 | 4177 - 4208 | pBR322_origin | + | GGTAACAG |
| PIM83 | 1 | -4 | GTTCACTGCCGTACAGGCAGCTTAGAAA | S206 | TTACGCATCTGTGCGGTATTTCACACCGCATA | 32 | 3614 - 3645 | bb | + | GGAGAAAA |
| PIM83 | 1 | -3 | GTTCACTGCCGTACAGGCAGCTTAGAAA | S207 | ATTCAGGTCGAGGTGGCCCGGCTCCATGCACC | 32 | 4667 - 4698 | bb | - | GGAAGCCG |
| PIM83 | 1 | -2 | GTTCACTGCCGTACAGGCAGCTTAGAAA | S208 | TTCGGAAAAAGAGTTGGTAGCTCTTGATCCGG | 32 | 4332 - 4363 | pBR322_origin | - | GGTAACTG |
| PIM83 | 1 | -1 | GTTCACTGCCGTACAGGCAGCTTAGAAA | S209 | GAACTGAGATACCTACAGCGTGAGCTATGAGA | 32 | 4041 - 4072 | pBR322_origin | + | GGTGTAGG |
| PIM84 | 1 | -3 | GTTCACTGCCGTACAGGCAGCTTAGAAA | S210 | ACGCTTCCCGAAGGGAGAAAGGCGGACAGGTA | 32 | 4002 - 4033 | pBR322_origin | + | GGCGCTTT |
| PIM84 | 1 | -2 | GTTCACTGCCGTACAGGCAGCTTAGAAA | S211 | ATTCAGGTCGAGGTGGCCCGGCTCCATGCACC | 32 | 4667 - 4698 | bb | - | GGAAGCCG |
| PIM84 | 1 | -1 | GTTCACTGCCGTACAGGCAGCTTAGAAA | S212 | TTCTAGTGTAGCCGTAGTTAGGCCACCACTTC | 32 | 4259 - 4290 | pBR322_origin | + | GGACAGTA |
| PIM85 | 1 | -2 | GTTCACTGCCGTACAGGCAGCTTAGAAA | S213 | AGAGCGCTGCCGGCACCTGTCCTACGAGTTGCA | 33 | 5163 - 5195 | bb | - | GGGTCATT |
| PIM85 | 1 | -1 | GTTCACTGCCGTACAGGCAGCTTAGAAA | S214 | GAACTGAGATACCTACAGCGTGAGCTATGAGA | 32 | 4041 - 4072 | pBR322_origin | + | GGTGTAGG |
| PIM86 | 1 | -1 | GTTCACTGCCGTACAGGCAGCTTAGAAA | S215 | GCAAGAATTGATTGGCTCCAATTCTTGGAGTG | 32 | 4603 - 4634 | bb | - | GGCCATGA |
| PIM87 | 1 | -3 | GTTCACTGCCGTACAGGCAGCTTAGAAA | S216 | AACCCGTTCCATGTGCTCGCCGAGGCGGCATAA | 33 | 4754 - 4786 | bb | - | GGCATGGA |
| PIM87 | 1 | -2 | GTTCACTGCCGTACAGGCAGCTTAGAAA | S217 | AAGGAAAGTCTACACGAACCCTTTGGCAAAAT | 32 | 3495 - 3526 | OriT | - | GGTGTATC |
| PIM87 | 1 | -1 | GTTCACTGCCGTACAGGCAGCTTAGAAA | S218 | TTTTTACGGTTCCTGGCCTTTTGCTGGCCTTT | 32 | 3801 - 3832 | bb | + | GGCCGCGT |
| PIM88 | 1 | -2 | GTTCACTGCCGTACAGGCAGCTTAGAAA | S219 | ATAGGCTCCGCCCCCCTGACGAGCATCACAAAA | 33 | 3856 - 3888 | pBR322_origin | - | GGAAAAAC |
| PIM88 | 1 | -1 | GTTCACTGCCGTACAGGCAGCTTAGAAA | S220 | TATCCTGCCCGGCTGACGCCGTTGGATACACC | 32 | 3463 - 3494 | OriT | - | GGTGAAGT |
| PIM89 | 1 | -1 | GTTCACTGCCGTACAGGCAGCTTAGAAA | S221 | TAACTACGGCTACACTAGAAGGACAGTATTTG | 32 | 4271 - 4302 | pBR322_origin | - | GGCCACCA |
| PIM90 | 1 | -2 | GTTCACTGCCGTACAGGCAGCTTAGAAA | S222 | AGCCTCGCGTCGCGAACGCCAGCAAGACGTAG | 32 | 4959 - 4990 | TcR | - | GGATGGCC |
| PIM90 | 1 | -1 | GTTCACTGCCGTACAGGCAGCTTAGAAA | S223 | TTCTAGTGTAGCCGTAGTTAGGCCACCACTTC | 32 | 4259 - 4290 | pBR322_origin | + | GGACAGTA |
| PIM91 | 1 | -4 | GTTCACTGCCGTACAGGCAGCTTAGAAA | S224 | GTTGATATATCCCAATGGCATCGTAAAGAACA | 32 | 1046 - 1077 | bb | - | GGTGGTAT |
| PIM91 | 1 | -3 | GTTCACTGCCGTACAGGCAGCTTAGAAA | S225 | TTGTCTGCCTCCCCGCGTTGCGTCGCGGTGCA | 32 | 4693 - 4724 | TcR | + | GGTATAGG |
| PIM91 | 1 | -2 | GTTCACTGCCGTACAGGCAGCTTAGAAA | S226 | CTTGTCTGCCTCCCCGCGTTGCGTCGCGGTGC | 32 | 4694 - 4725 | TcR | + | GTATAGGG |
| PIM91 | 1 | -1 | GTTCACTGCCGTACAGGCAGCTTAGAAA | S227 | ATTCAGGTCGAGGTGGCCCGGCTCCATGCACC | 32 | 4667 - 4698 | TcR | - | GGAAGCCG |
| PIM92 | 1 | -2 | GTTCACTGCCGTACAGGCAGCTTAGAAA | S228 | CCGATGCCGCCGGAAGCGAGAAGAATCATAATG | 33 | 4913 - 4945 | TcR | - | GATGCCCG |
| PIM92 | 1 | -1 | GTTCACTGCCGTACAGGCAGCTTAGAAA | S229 | AGTGGCGATAAGTCGTGTCTTACCGGGTTGGAC | 33 | 4163 - 4195 | pBR322_origin | + | GGCAGCAG |

^a^+/- refers to the protospacer targeted strand on the plasmid.

^b^PAM is defined as 5’-protospacer-PAM-3’ on the targeted strand (8 nt of flanking sequence is shown).

**Table S5.** pPF575 (+ strand primed protospacer) PIMs derived from WT *P. atrosepticum*

| **PIM** | **CRISPR locus** | **spacer position** | **repeat (5'-3')-28bp** | **spacer #** | **spacer (5'-3')** | **spacer (nt)** | **target nt** | **target** | **-/+^a^** | **PAM^b^** |
| --- | --- | --- | --- | --- | --- | --- | --- | --- | --- | --- |
| **WT** | **1** | **1** | **GTTCACTGCCGTACAGGCAGCTTAGAAA** | **C1S1** | **GCAGAGACTATCGATACGGTCTGGACGGATGC** | **32** | **4516 - 4550** |  | **+** | **TGCCCATG** |
| PIM93 | 1 | -4 | GTTCACTGCCGTACAGGCAGCTTAGAAA | S230 | TACACCGAACTGAGATACCTACAGCGTGAGCT | 32 | 4047 - 4078 | pBR322_origin | + | GGTCGTTC |
| PIM93 | 1 | -3 | GTTCACTGCCGTACAGGCAGCTTAGAAA | S231 | AGACCGTATCGATAGTCTCTGCTGTGTCTGAC | 32 | 4526 - 4557 | TcR | - | GGACGGAT |
| PIM94 | 1 | -1 | GTTCACTGCCGTACAGGCAGCTTAGAAA | S232 | ACCAAACGTTTCGGCGAGAAGCAGGCCATTAT | 32 | 5018 - 5049 | TcR | + | GGCGGGAC |
| PIM95 | 1 | -2 | GTTCACTGCCGTACAGGCAGCTTAGAAA | S233 | GCCTGGCCCTGAGAGAGTTGCAGCAAGCGGTC | 32 | 2067 - 2098 | lacI | - | GGTGAAGG |
| PIM95 | 1 | -1 | GTTCACTGCCGTACAGGCAGCTTAGAAA | S234 | ATTGGGATATATCAACGGTGGTATATCCAGTG | 32 | 1030 - 1061 | bb | + | GGCATCGT |
| PIM95 | 2 | -1 | GTTCACTGCCGTACAGGCAGCTTAGAAA | S235 | TGAATCGCCAGCGGCATCAGCACCTTGTCGCC | 32 | 1553 - 1584 | bb | + | GGTTCATC |
| PIM96 | 1 | -1 | GTTCACTGCCGTACAGGCAGCTTAGAAA | S236 | ACCGGAAGGAGCTGACTGGGTTGAAGGCTCTCA | 33 | 5247 - 5279 | TcR | - | GGGCGCGG |
| PIM97 | 1 | -1 | GTTCACTGCCGTACAGGCAGCTTAGAAA | S237 | TTCTAGTGTAGCCGTAGTTAGGCCACCACTTC | 32 | 4259 - 4290 | pBR322_origin | + | GGACAGTA |
| PIM98 | 1 | -3 | GTTCACTGCCGTACAGGCAGCTTAGAAA | S238 | GCATAGTTAAGCCAGTATACACTCCGCTATCG | 32 | 3295 - 3326 | bb | + | GGCATCAG |
| PIM98 | 1 | -2 | GTTCACTGCCGTACAGGCAGCTTAGAAA | S239 | TTGGAGCCGTACATGAACTGAGGGGACAGGAT | 32 | 312 - 343 | mCherry | + | GGCCTACG |
| PIM98 | 1 | -1 | GTTCACTGCCGTACAGGCAGCTTAGAAA | S240 | TGACGAGCATCACAAAAATCGACGCTCAAGTC | 32 | 3872 - 3903 | pBR322_origin | - | GGGGGGCG |
| PIM98 | 2 | -1 | GTTCACTGCCGTACAGGCAGCTTAGAAA | S241 | TTACGCATCTGTGCGGTATTTCACACCGCATA | 32 | 3614 - 3645 | bb | + | GGAGAAAA |
| PIM99 | 1 | -1 | GTTCACTGCCGTACAGGCAGCTTAGAAA | S242 | CAGAGCGCTGCCGGCACCTGTCCTACGAGTTG | 32 | 5162 - 5193 | TcR | - | GGTCATTT |
| PIM100 | 1 | -1 | GTTCACTGCCGTACAGGCAGCTTAGAAA | S243 | GTGACGATCAGCGGTCCAATGATCGAAGTTAG | 32 | 4793 - 4824 | TcR | - | GGCGATTT |
| PIM100 | 2 | -1 | GTTCACTGCCGTACAGGCAGCTTAGAAA | S244 | GAAGGTAACTGGCTTCAGCAGAGCGCAGATAC | 32 | 4303 - 4334 | pBR322_origin | + | GGAAAAAG |
| PIM101 | 1 | -6 | GTTCACTGCCGTACAGGCAGCTTAGAAA | S245 | TGCCTGGACAGCATGGCCTGCAACGCGGGCAT | 32 | 4880 - 4911 | TcR | - | GGTAGATG |
| PIM101 | 1 | -5 | GTTCACTGCCGTACAGGCAGCTTAGAAA | S246 | ATACCCACGCCGAAACAAGCGCTCATGAGCCC | 32 | 5429 - 5460 | TcR | - | GGTGGCAG |
| PIM101 | 1 | -4 | GTTCACTGCCGTACAGGCAGCTTAGAAA | S247 | GCTACCAGCGGTGGTTTGTTTGCCGGATCAAG | 32 | 4354 - 4385 | pBR322_origin | + | GGTGGTTT |
| PIM101 | 1 | -3 | GTTCACTGCCGTACAGGCAGCTTAGAAA | S248 | TTCCGGTGGGCGCGGGGCATGACTATCGTCGC | 32 | 5222 - 5253 | TcR | + | GGAGCTGA |
| PIM101 | 1 | -2 | GTTCACTGCCGTACAGGCAGCTTAGAAA | S249 | TCGCGTCGCGAACGCCAGCAAGACGTAGCCCA | 32 | 4963 - 4994 | TcR | - | GGCTGGAT |
| PIM101 | 1 | -1 | GTTCACTGCCGTACAGGCAGCTTAGAAA | S250 | GCTACCAGCGGTGGTTTGTTTGCCGGATCAAG | 32 | 4354 - 4385 | pBR322_origin | + | GGTGGTTT |
| PIM101 | 2 | -1 | GTTCACTGCCGTACAGGCAGCTTAGAAA | S251 | GTATACACTCCGCTATCGCTACGTGACTGGGT | 32 | 3281 - 3312 | bb | + | TGGCTTAA |
| PIM102 | 1 | -1 | GTTCACTGCCGTACAGGCAGCTTAGAAA | S252 | ATGCACCGCGACGCAACGCGGGGAGGCAGACA | 32 | 4692 - 4723 | TcR | - | GGAGCCGG |
| PIM103 | 1 | -1 | GTTCACTGCCGTACAGGCAGCTTAGAAA | S253 | ATGCACCGCGACGCAACGCGGGGAGGCAGACA | 32 | 4692 - 4723 | TcR | - | GGAGCCGG |
| PIM104 | 1 | -2 | GTTCACTGCCGTACAGGCAGCTTAGAAA | S254 | TCGCTAACGGATTCACCACTCCAAGAATTGGA | 32 | 4619 - 4650 | TcR | + | GGTGCCGC |
| PIM104 | 1 | -1 | GTTCACTGCCGTACAGGCAGCTTAGAAA | S255 | TACGTGAAGCACCCCGCCGACATCCCCGACTAC | 33 | 348 - 380 | mCherry | - | GGCCTTGG |
| PIM104 | 2 | -1 | GTTCACTGCCGTACAGGCAGCTTAGAAA | S256 | CGTTCCATGTGCTCGCCGAGGCGGCATAAATC | 32 | 4758 - 4789 | TcR | - | GGTTGGCA |
| PIM105 | 1 | -2 | GTTCACTGCCGTACAGGCAGCTTAGAAA | S257 | CGTTAGCGAGGTGCCGCCGGCTTCCATTCAGGT | 33 | 4642 - 4674 | TcR | - | GATTCACC |
| PIM105 | 1 | -1 | GTTCACTGCCGTACAGGCAGCTTAGAAA | S258 | TGCAACGCGGGCATCCCGATGCC**R**CCGGAAGCG | 33 | 4898 - 4930 | TcR | - | GGCCATGC |
| PIM105 | 2 | -1 | GTTCACTGCCGTACAGGCAGCTTAGAAA | S259 | TGTCCTACGAGTTGCATGATAAAGAAGACAGT | 32 | 5180 - 5211 | TcR | - | GGTGCCGG |
| PIM106 | 2 | -1 | GTTCACTGCCGTACAGGCAGCTTAGAAA | S260 | TCGTAGGGGCGGCCCTCGCCCTCGCCCTCGAT | 32 | 219 - 250 | mCherry | + | GGGCACCC |
| PIM107 | 1 | -2 | GTTCACTGCCGTACAGGCAGCTTAGAAA | S261 | TGGACAGCATGGCCTGCAACGCGGGCATCCCG | 32 | 4884 - 4915 | TcR | - | GGCAGGTA |
| PIM107 | 1 | -1 | GTTCACTGCCGTACAGGCAGCTTAGAAA | S262 | AGCCTCGCGTCGCGAACGCCAGCAAGACGTAG | 32 | 4959 - 4990 | TcR | - | GGATGGCC |
| PIM108 | 1 | -3 | GTTCACTGCCGTACAGGCAGCTTAGAAA | S263 | ACTTCAAGAACTCTGTAGCACCGCCTACATAC | 32 | 4232 - 4263 | pBR322_origin | + | GGTGGCCT |
| PIM108 | 1 | -2 | GTTCACTGCCGTACAGGCAGCTTAGAAA | S264 | GGCGCCACAGGTGCGGTTGCTGGCGCCTATATC | 33 | 5500 - 5532 | TcR | + | GGTGATGC |
| PIM108 | 1 | -1 | GTTCACTGCCGTACAGGCAGCTTAGAAA | S265 | CGCCAAGCTGAAGGTGACCAAGGGTGGCCCCCT | 33 | 263 - 295 | mCherry | - | GTCTGGGT |
| PIM108 | 2 | -3 | GTTCACTGCCGTACAGGCAGCTTAGAAA | S266 | TACAGCATCCAGGGTGACGGTGCCGAGGATGA | 32 | 5803 - 5834 | TcR | - | GGCATAGG |
| PIM108 | 2 | -2 | GTTCACTGCCGTACAGGCAGCTTAGAAA | S267 | TACTACTGGGCTGCTTCCTAATGCAGGAGTCG | 32 | 5308 - 5339 | TcR | + | GGTTGAGG |
| PIM108 | 2 | -1 | GTTCACTGCCGTACAGGCAGCTTAGAAA | S268 | GGTGGGCGCGGGGCATGACTATCGTCGCCGCA | 32 | 5218 - 5249 | TcR | + | GGAAGGAG |
| PIM109 | 1 | -3 | GTTCACTGCCGTACAGGCAGCTTAGAAA | S269 | AACCGCGTGGCACAACAACTGGCGGGCAAACA | 32 | 2823 - 2854 | lacI | + | GGGAATGT |
| PIM109 | 1 | -2 | GTTCACTGCCGTACAGGCAGCTTAGAAA | S270 | TGAATGGAAGCCGGCGGCACCTCGCTAACGGA | 32 | 4640 - 4671 | TcR | + | GGTCGAGG |

^a^+/- refers to the protospacer targeted strand on the plasmid.

^b^PAM is defined as 5’-protospacer-PAM-3’ on the targeted strand (8 nt of flanking sequence is shown).

**Table S5 continued.** pPF575 (+ strand primed protospacer) PIMs derived from WT *P. atrosepticum*

| **PIM** | **CRISPR locus** | **spacer position** | **repeat (5'-3')-28bp** | **spacer #** | **spacer (5'-3')** | **spacer (nt)** | **target nt** | **target** | **-/+^a^** | **PAM^b^** |
| --- | --- | --- | --- | --- | --- | --- | --- | --- | --- | --- |
| PIM109 | 1 | -1 | GTTCACTGCCGTACAGGCAGCTTAGAAA | S271 | GATAGCGGAACGGGAAGGCGACTGGAGTGCCA | 32 | 2335 - 2366 | lacI | + | GGCTGAAT |
| PIM109 | 2 | -1 | GTTCACTGCCGTACAGGCAGCTTAGAAA | S272 | TGCCTGGACAGCATGGCCTGCAACGCGGGCAT | 32 | 4880 - 4911 | TcR | - | GGTAGATG |
| PIM110 | 1 | -4 | GTTCACTGCCGTACAGGCAGCTTAGAAA | S273 | TCGCGTCGCGAACGCCAGCAAGACGTAGCCCA | 32 | 4963 - 4994 | TcR | - | GGCTGGAT |
| PIM110 | 1 | -3 | GTTCACTGCCGTACAGGCAGCTTAGAAA | S274 | TCGACCTGAATGGAAGCCGGCGGCACCTCGCTA | 33 | 4645 - 4677 | TcR | + | GGTGGCCC |
| PIM110 | 1 | -2 | GTTCACTGCCGTACAGGCAGCTTAGAAA | S275 | AGGGGGAAACGCCTGGTATCTTTATAGTCCTG | 32 | 3922 - 3953 | pBR322_origin | + | GGAAGCTC |
| PIM110 | 2 | -2 | GTTCACTGCCGTACAGGCAGCTTAGAAA | S276 | AGGTGCGAATAAGGGACAGTGAAGAAGGAACAC | 33 | 3404 - 3436 | RP4/OriT | - | GGCGGTGC |
| PIM110 | 2 | -1 | GTTCACTGCCGTACAGGCAGCTTAGAAA | S277 | TTGTCTGCCTCCCCGCGTTGCGTCGCGGTGCA | 32 | 4693 - 4724 | TcR | + | GGTATAGG |
| PIM111 | 1 | -1 | GTTCACTGCCGTACAGGCAGCTTAGAAA | S278 | TCGCTCTGCTAATCCTGTTACCAGTGGCTGCT | 32 | 4199 - 4230 | pBR322_origin | + | GGTATGTA |
| PIM111 | 2 | -2 | GTTCACTGCCGTACAGGCAGCTTAGAAA | S279 | AGCGGTGGTTTGTTTGCCGGATCAAGAGCTAC | 32 | 4348 - 4379 | pBR322_origin | + | GGTAGCGG |
| PIM112 | 1 | -4 | GTTCACTGCCGTACAGGCAGCTTAGAAA | S280 | TAGAAAAGATCAAAGGATCTTCTTGAGATCCTT | 33 | 4429 - 4461 | bb | + | CGGGGTCT |
| PIM112 | 1 | -3 | GTTCACTGCCGTACAGGCAGCTTAGAAA | S281 | GGATCAAGAGCTACCAACTCTTTTTCCGAAGG | 32 | 4330 - 4361 | pBR322_origin | + | GGCAAACA |
| PIM112 | 1 | -2 | GTTCACTGCCGTACAGGCAGCTTAGAAA | S282 | TCGACCTGAATGGAAGCCGGCGGCACCTCGCT | 32 | 4646 - 4677 | TcR | + | GGTGGCCC |
| PIM112 | 1 | -1 | GTTCACTGCCGTACAGGCAGCTTAGAAA | S283 | AGCTTGGAGCGAACGACCTACACCGAACTGAG | 32 | 4065 - 4096 | pBR322_origin | + | GGGCTGTG |
| PIM112 | 2 | -1 | GTTCACTGCCGTACAGGCAGCTTAGAAA | S284 | ATGTGCTCGCCGAGGCGGCATAAATCGCCGTG | 32 | 4764 - 4795 | TcR | - | GGAACGGG |
| PIM113 | 2 | -1 | GTTCACTGCCGTACAGGCAGCTTAGAAA | S285 | CTGCAACGCGGGCATCCCGATGCCGCCGGAAGC | 33 | 4897 - 4929 | TcR | - | GCCATGCT |
| PIM114 | 2 | -1 | GTTCACTGCCGTACAGGCAGCTTAGAAA | S286 | TCGCCGAAAATGACCCAGAGCGCTGCCGGCAC | 32 | 5147 - 5178 | TcR | - | GGACCGCT |
| PIM115 | 1 | -2 | GTTCACTGCCGTACAGGCAGCTTAGAAA | S287 | ACGGGGCCTGCCACCATACCCACGCCGAAACAA | 33 | 5414 - 5446 | TcR | - | GGCCGGGG |
| PIM115 | 2 | -1 | GTTCACTGCCGTACAGGCAGCTTAGAAA | S288 | TCGACCTGAATGGAAGCCGGCGGCACCTCGCT | 32 | 4646 - 4677 | TcR | + | GGTGGCCC |
| PIM116 | 1 | -3 | GTTCACTGCCGTACAGGCAGCTTAGAAA | S289 | GGCTTCCATTCAGGTCGAGGTGGCCCGGCTCCA | 33 | 4660 - 4692 | TcR | - | GGCGGCAC |
| PIM117 | 1 | -1 | GTTCACTGCCGTACAGGCAGCTTAGAAA | S290 | GAATGGAAGCCGGCGGCACCTCGCTAACGGAT | 32 | 4639 - 4670 | TcR | + | AGGTCGAG |
| PIM117 | 2 | -2 | GTTCACTGCCGTACAGGCAGCTTAGAAA | S291 | AGCCTAACTTCGATCATTGGACCGCTGATCGT | 32 | 4796 - 4827 | TcR | + | GGTAAGAG |
| PIM117 | 2 | -1 | GTTCACTGCCGTACAGGCAGCTTAGAAA | S292 | AGTGGCTGCTGCCAGTGGCGATAAGTCGTGTC | 32 | 4177 - 4208 | pBR322_origin | + | GGTAACAG |
| PIM118 | 1 | -1 | GTTCACTGCCGTACAGGCAGCTTAGAAA | S293 | ACCGGAAGGAGCTGACTGGGTTGAAGGCTCTCAA | 34 | 5247 - 5280 | TcR | - | GGGCGCGG |
| PIM119 | 1 | -3 | GTTCACTGCCGTACAGGCAGCTTAGAAA | S294 | CGCTGATCGTCACGGCGATTTATGCCGCCTCG | 32 | 4774 - 4805 | TcR | + | GTCCAATG |
| PIM119 | 1 | -2 | GTTCACTGCCGTACAGGCAGCTTAGAAA | S295 | TGTCCTACGAGTTGCATGATAAAGAAGACAGT | 32 | 5180 - 5211 | TcR | - | GGTGCCGG |
| PIM119 | 1 | -1 | GTTCACTGCCGTACAGGCAGCTTAGAAA | S296 | TTTCGCGGTATGGCATGATAGCGCCCGGAAGA | 32 | 3024 - 3055 | bb | + | GGTTTTGC |
| PIM120 | 1 | -3 | GTTCACTGCCGTACAGGCAGCTTAGAAA | S297 | AGCGGTGGTTTGTTTGCCGGATCAAGAGCTAC | 32 | 4348 - 4379 | pBR322_origin | + | GGTAGCGG |
| PIM120 | 1 | -2 | GTTCACTGCCGTACAGGCAGCTTAGAAA | S298 | TCGCGTCGCGAACGCCAGCAAGACGTAGCCCA | 32 | 4963 - 4994 | TcR | - | GGCTGGAT |
| PIM120 | 1 | -1 | GTTCACTGCCGTACAGGCAGCTTAGAAA | S299 | TTATGGTGAGCAAGGGCGAGGAGGATAACATG | 32 | 118 - 149 | bb | - | GGGACTCC |
| PIM120 | 2 | -1 | GTTCACTGCCGTACAGGCAGCTTAGAAA | S300 | GTATCGATAGTCTCTGCTGTGTCTGACGCTCA | 32 | 4531 - 4562 | TcR | - | GGTCTGGA |
| PIM121 | 2 | -3 | GTTCACTGCCGTACAGGCAGCTTAGAAA | S301 | GAAGCAGGGTTATGCAGCGGAAAAGCGCTCAT | 32 | 3583 - 3614 | bb | - | GGGGTCAT |
| PIM121 | 2 | -2 | GTTCACTGCCGTACAGGCAGCTTAGAAA | S302 | AGACACCCATCAACAGTATTATTTTCTCCCAT | 32 | 2513 - 2544 | lacI | + | GGTCAGAG |
| PIM121 | 2 | -1 | GTTCACTGCCGTACAGGCAGCTTAGAAA | S303 | ACAACGAGGACTACACCATCGTGGAACAGTAC | 32 | 745 - 776 | mCherry | - | GGGAGGTG |
| PIM122 | 1 | -2 | GTTCACTGCCGTACAGGCAGCTTAGAAA | S304 | CAGAGCGCTGCCGGCACCTGTCCTACGAGTTG | 32 | 5162 - 5193 | TcR | - | GGTCATTT |
| PIM122 | 1 | -1 | GTTCACTGCCGTACAGGCAGCTTAGAAA | S305 | ATTCAGGTCGAGGTGGCCCGGCTCCATGCACC | 32 | 4667 - 4698 | TcR | - | GGAAGCCG |
| PIM122 | 2 | -1 | GTTCACTGCCGTACAGGCAGCTTAGAAA | S306 | AACCGCGTGGCACAACAACTGGCGGGCAAACA | 32 | 2823 - 2854 | lacI | + | GGGAATGT |
| PIM123 | 1 | -1 | GTTCACTGCCGTACAGGCAGCTTAGAAA | S307 | AGCTTGGAGCGAACGACCTACACCGAACTGAG | 32 | 4065 - 4096 | pBR322_origin | + | GGGCTGTG |
| PIM124 | 1 | -2 | GTTCACTGCCGTACAGGCAGCTTAGAAA | S308 | TGAATGGAAGCCGGCGGCACCTCGCTAACGGA | 32 | 4640 - 4671 | TcR | + | GGTCGAGG |
| PIM124 | 1 | -1 | GTTCACTGCCGTACAGGCAGCTTAGAAA | S309 | TGATGGTCGTCATCTACCTGCCTGGACAGCAT | 32 | 4862 - 4893 | TcR | - | GGGACAGC |
| PIM125 | 1 | -1 | GTTCACTGCCGTACAGGCAGCTTAGAAA | S310 | ATGAGCAAACTGAAACGTTTTCATCGCTCTGG | 32 | 1302 - 1333 | bb | - | GGAAAACG |
| PIM125 | 2 | -1 | GTTCACTGCCGTACAGGCAGCTTAGAAA | S311 | TCAGCGTCGTAGTGGCCGCCGTCCTTCAGCTT | 32 | 630 - 661 | mCherry | + | GGTCAAGA |
| PIM126 | 1 | -2 | GTTCACTGCCGTACAGGCAGCTTAGAAA | S312 | TTCCGGTGGGCGCGGGGCATGACTATCGTCGC | 32 | 5222 - 5253 | TcR | + | GGAGCTGA |

^a^+/- refers to the protospacer targeted strand on the plasmid.

^b^PAM is defined as 5’-protospacer-PAM-3’ on the targeted strand (8 nt of flanking sequence is shown).

**Table S5 continued.** pPF575 (+ strand primed protospacer) PIMs derived from WT *P. atrosepticum*

| **PIM** | **CRISPR locus** | **spacer position** | **repeat (5'-3')-28bp** | **spacer #** | **spacer (5'-3')** | **spacer (nt)** | **target nt** | **target** | **-/+^a^** | **PAM^b^** |
| --- | --- | --- | --- | --- | --- | --- | --- | --- | --- | --- |
| PIM126 | 1 | -1 | GTTCACTGCCGTACAGGCAGCTTAGAAA | S313 | GCAAGGAATGGTGCATGCAAGGAGATGGCGCC | 32 | 5366 - 5397 | TcR | - | GGCGGCGG |
| PIM126 | 2 | -1 | GTTCACTGCCGTACAGGCAGCTTAGAAA | S314 | AGGCAGGTAGATGACGACCATCAGGGACAGCT | 32 | 4853 - 4884 | TcR | + | GGACAGCA |
| PIM127 | 1 | -1 | GTTCACTGCCGTACAGGCAGCTTAGAAA | S315 | AAATACTGTCCTTCTAGTGTAGCCGTAGTTAG | 32 | 4270 - 4301 | pBR322_origin | + | GGTATCTG |
| PIM128 | 1 | -3 | GTTCACTGCCGTACAGGCAGCTTAGAAA | S316 | GTGGCCAGGACCCAACGCTGCCCGAAATTCCG | 32 | 3079 - 3110 | bb | + | GGGTGCGC |
| PIM128 | 1 | -2 | GTTCACTGCCGTACAGGCAGCTTAGAAA | S317 | GCTACCAGCGGTGGTTTGTTTGCCGGATCAAG | 32 | 4354 - 4385 | pBR322_origin | + | GGTGGTTT |
| PIM128 | 1 | -1 | GTTCACTGCCGTACAGGCAGCTTAGAAA | S318 | TGGACAGCATGGCCTGCAACGCGGGCATCCCGA | 33 | 4884 - 4916 | TcR | - | GGCAGGTA |
| PIM128 | 2 | -1 | GTTCACTGCCGTACAGGCAGCTTAGAAA | S319 | CTGGTAACAGGATTAGCAGAGCGAGGTATGTA | 32 | 4207 - 4238 | pBR322_origin | - | TGGCTGCT |
| PIM129 | 1 | -3 | GTTCACTGCCGTACAGGCAGCTTAGAAA | S320 | TGCCTGGACAGCATGGCCTGCAACGCGGGCAT | 32 | 4880 - 4911 | TcR | - | GGTAGATG |
| PIM129 | 1 | -2 | GTTCACTGCCGTACAGGCAGCTTAGAAA | S321 | AGCTTGGAGCGAACGACCTACACCGAACTGAG | 32 | 4065 - 4096 | pBR322_origin | + | GGGCTGTG |
| PIM129 | 1 | -1 | GTTCACTGCCGTACAGGCAGCTTAGAAA | S322 | TTCTAGTGTAGCCGTAGTTAGGCCACCACTTC | 32 | 4259 - 4290 | pBR322_origin | + | GGACAGTA |
| PIM129 | 2 | -1 | GTTCACTGCCGTACAGGCAGCTTAGAAA | S323 | ACCCTGAATTGACTCTCTTCCGGGCGCTATCA | 32 | 3009 - 3040 | bb | - | GGTGAATG |
| PIM130 | 2 | -1 | GTTCACTGCCGTACAGGCAGCTTAGAAA | S324 | TGTTGATAGATCCAGTAATGACCTCAGAACTC | 32 | 884 - 915 | bb | - | GGAGTCCA |
| PIM131 | 1 | -1 | GTTCACTGCCGTACAGGCAGCTTAGAAA | S325 | ATGCACCGCGACGCAACGCGGGGAGGCAGACA | 32 | 4692 - 4723 | TcR | - | GGAGCCGG |
| PIM131 | 2 | -2 | GTTCACTGCCGTACAGGCAGCTTAGAAA | S326 | TCGCCGAAAATGACCCAGAGCGCTGCCGGCAC | 32 | 5147 - 5178 | TcR | - | GGACCGCT |
| PIM131 | 2 | -1 | GTTCACTGCCGTACAGGCAGCTTAGAAA | S327 | AGCTTGGAGCGAACGACCTACACCGAACTGAGA | 33 | 4064 - 4096 | pBR322_origin | + | GGGCTGTG |
| PIM132 | 1 | -1 | GTTCACTGCCGTACAGGCAGCTTAGAAA | S328 | TATTTCCCTAAAGGGTTTATTGAGAATATGTT | 32 | 1412 - 1443 | bb | - | GGCCAGGT |
| PIM133 | 2 | -1 | GTTCACTGCCGTACAGGCAGCTTAGAAA | S329 | ACGCTTCCCGAAGGGAGAAAGGCGGACAGGTA | 32 | 4002 - 4033 | pBR322_origin | + | GGCGCTTT |
| PIM134 | 1 | -4 | GTTCACTGCCGTACAGGCAGCTTAGAAA | S330 | AGTGGCGATAAGTCGTGTCTTACCGGGTTGGA | 32 | 4164 - 4195 | pBR322_origin | + | GGCAGCAG |
| PIM134 | 1 | -3 | GTTCACTGCCGTACAGGCAGCTTAGAAA | S331 | CGAAGCAGGGTTATGCAGCGGAAAAGCGCTCA | 32 | 3582 - 3613 | bb | - | GGGTCATT |
| PIM134 | 1 | -2 | GTTCACTGCCGTACAGGCAGCTTAGAAA | S332 | TTACGCATCTGTGCGGTATTTCACACCGCATA | 32 | 3614 - 3645 | bb | + | GGAGAAAA |
| PIM134 | 1 | -1 | GTTCACTGCCGTACAGGCAGCTTAGAAA | S333 | ATTCAGGTCGAGGTGGCCCGGCTCCATGCACC | 32 | 4667 - 4698 | TcR | - | GGAAGCCG |
| PIM134 | 2 | -1 | GTTCACTGCCGTACAGGCAGCTTAGAAA | S334 | TTCTAGTGTAGCCGTAGTTAGGCCACCACTTC | 32 | 4259 - 4290 | pBR322_origin | + | GGACAGTA |
| PIM135 | 2 | -3 | GTTCACTGCCGTACAGGCAGCTTAGAAA | S335 | GGATCAAGAGCTACCAACTCTTTTTCCGAAGG | 32 | 4330 - 4361 | pBR322_origin | + | GGCAAACA |
| PIM135 | 2 | -2 | GTTCACTGCCGTACAGGCAGCTTAGAAA | S336 | TATGCCTACAGCATCCAGGGTGACGGTGCCGA | 32 | 5797 - 5828 | TcR | - | GGCTTGGT |
| PIM135 | 2 | -1 | GTTCACTGCCGTACAGGCAGCTTAGAAA | S337 | CGTGGCCGGGGGACTGTTGGGCGCCATCTCCT | 32 | 5385 - 5416 | TcR | + | GGGCCTGC |
| PIM136 | 1 | -2 | GTTCACTGCCGTACAGGCAGCTTAGAAA | S338 | TTGAAGCTGTCCCTGATGGTCGTCATCTACCT | 32 | 4849 - 4880 | TcR | - | GGATCGCT |
| PIM136 | 1 | -1 | GTTCACTGCCGTACAGGCAGCTTAGAAA | S339 | CGTTCCATGTGCTCGCCGAGGCGGCATAAATC | 32 | 4758 - 4789 | TcR | - | GGTTGGCA |
| PIM137 | 1 | -3 | GTTCACTGCCGTACAGGCAGCTTAGAAA | S340 | GCGGTGGTTTGTTTGCCGGATCAAGAGCTACC | 32 | 4347 - 4378 | pBR322_origin | + | TGGTAGCG |
| PIM137 | 1 | -2 | GTTCACTGCCGTACAGGCAGCTTAGAAA | S341 | CGAAGGGAGAAAGGCGGACAGGTATCCGGTAA | 32 | 3994 - 4025 | pBR322_origin | + | GGAAGCGT |
| PIM137 | 1 | -1 | GTTCACTGCCGTACAGGCAGCTTAGAAA | S342 | AGTAGTAGGTTGAGGCCGTTGAGCACCGCCGC | 32 | 5333 - 5364 | TcR | - | GGGCTGCT |
| PIM137 | 2 | -1 | GTTCACTGCCGTACAGGCAGCTTAGAAA | S343 | TCGCTCAAGCCTTCGTCACTGGTCCCGCCACC | 32 | 5046 - 5077 | TcR | + | GGGCGTGC |
| PIM138 | 2 | -2 | GTTCACTGCCGTACAGGCAGCTTAGAAA | S344 | AACCCGGTAAGACACGACTTATCGCCACTGGC | 32 | 4167 - 4198 | pBR322_origin | - | GGACTCAA |
| PIM138 | 2 | -1 | GTTCACTGCCGTACAGGCAGCTTAGAAA | S345 | AGGCGTTTCCCCCTGGAAGCTCCCTCGTGCGC | 32 | 3940 - 3971 | pBR322_origin | - | GGTATCTT |
| PIM139 | 1 | -3 | GTTCACTGCCGTACAGGCAGCTTAGAAA | S346 | CACTGCGATGCTGGTTGCCAACGATCAGATGG | 32 | 2254 - 2285 | lacI | + | GGAACGAT |
| PIM139 | 1 | -2 | GTTCACTGCCGTACAGGCAGCTTAGAAA | S347 | TCCAGTCTGGCCCTGCACGCGCCGTCGCAAAT | 32 | 2766 - 2797 | lacI | + | GGTGGCAA |
| PIM139 | 1 | -1 | GTTCACTGCCGTACAGGCAGCTTAGAAA | S348 | TGACGAGCATCACAAAAATCGACGCTCAAGTC | 32 | 3872 - 3903 | pBR322_origin | - | GGGGGGCG |
| PIM140 | 1 | -3 | GTTCACTGCCGTACAGGCAGCTTAGAAA | S349 | TTCTAGTGTAGCCGTAGTTAGGCCACCACTTC | 32 | 4259 - 4290 | pBR322_origin | + | GGACAGTA |
| PIM140 | 1 | -2 | GTTCACTGCCGTACAGGCAGCTTAGAAA | S350 | GGATAAGGCGCAGCGGTCGGGCTGAACGGGGG | 32 | 4114 - 4145 | pBR322_origin | + | GGTAACTA |
| PIM140 | 1 | -1 | GTTCACTGCCGTACAGGCAGCTTAGAAA | S351 | AGCCTCGCGTCGCGAACGCCAGCAAGACGTAG | 32 | 4959 - 4990 | TcR | - | GGATGGCC |

^a^+/- refers to the protospacer targeted strand on the plasmid.

^b^PAM is defined as 5’-protospacer-PAM-3’ on the targeted strand (8 nt of flanking sequence is shown).


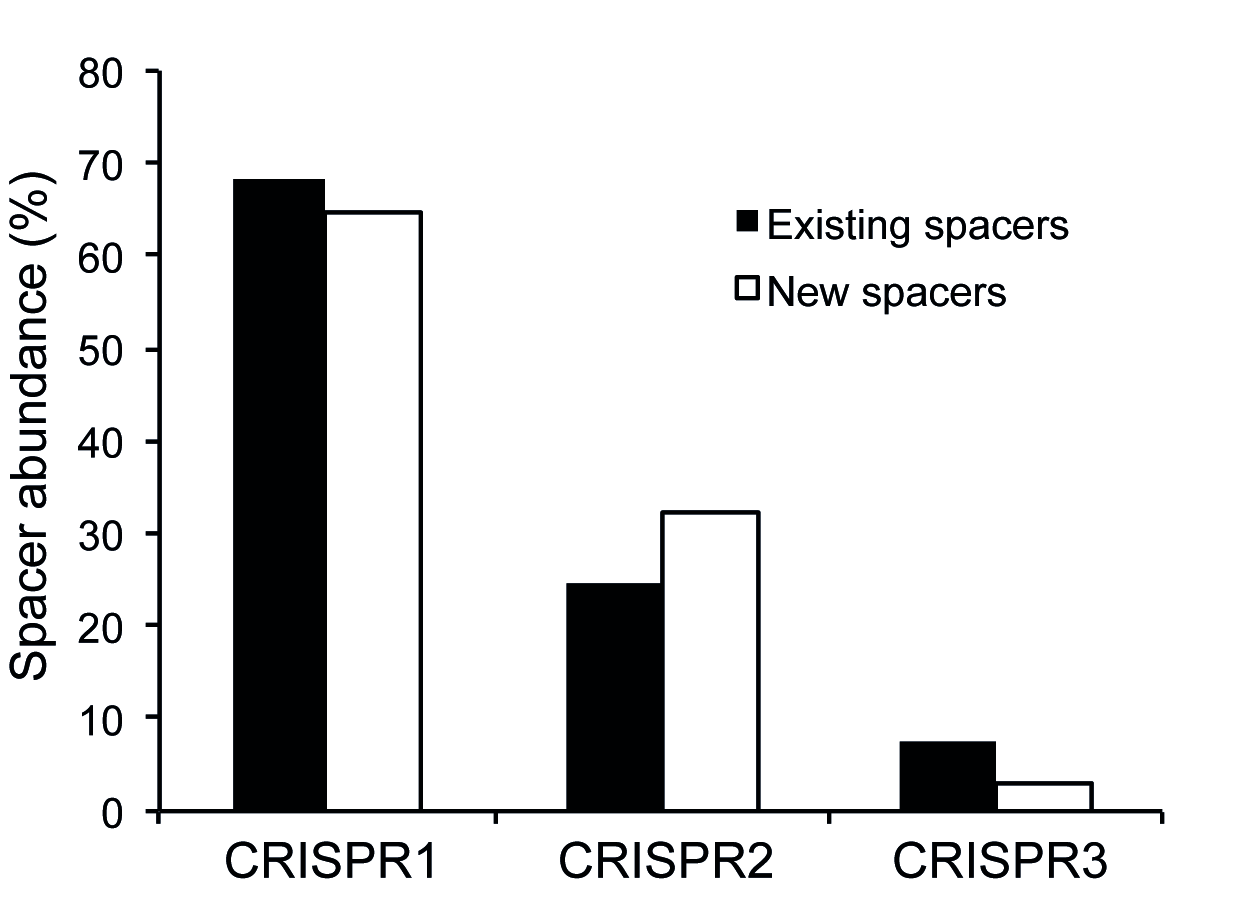


**Figure S1.** The array distribution of newly acquired spacers is similar to the distribution of original spacers in CRISPR arrays 1, 2 and 3. The percentage of total existing spacers per array is shown in black bars (28, 10 and 3 spacers in CRISPR1, 2 and 3, respectively) and compared with the percentage of newly acquired spacers found within each CRISPR array (white bars). See also Figure 2.


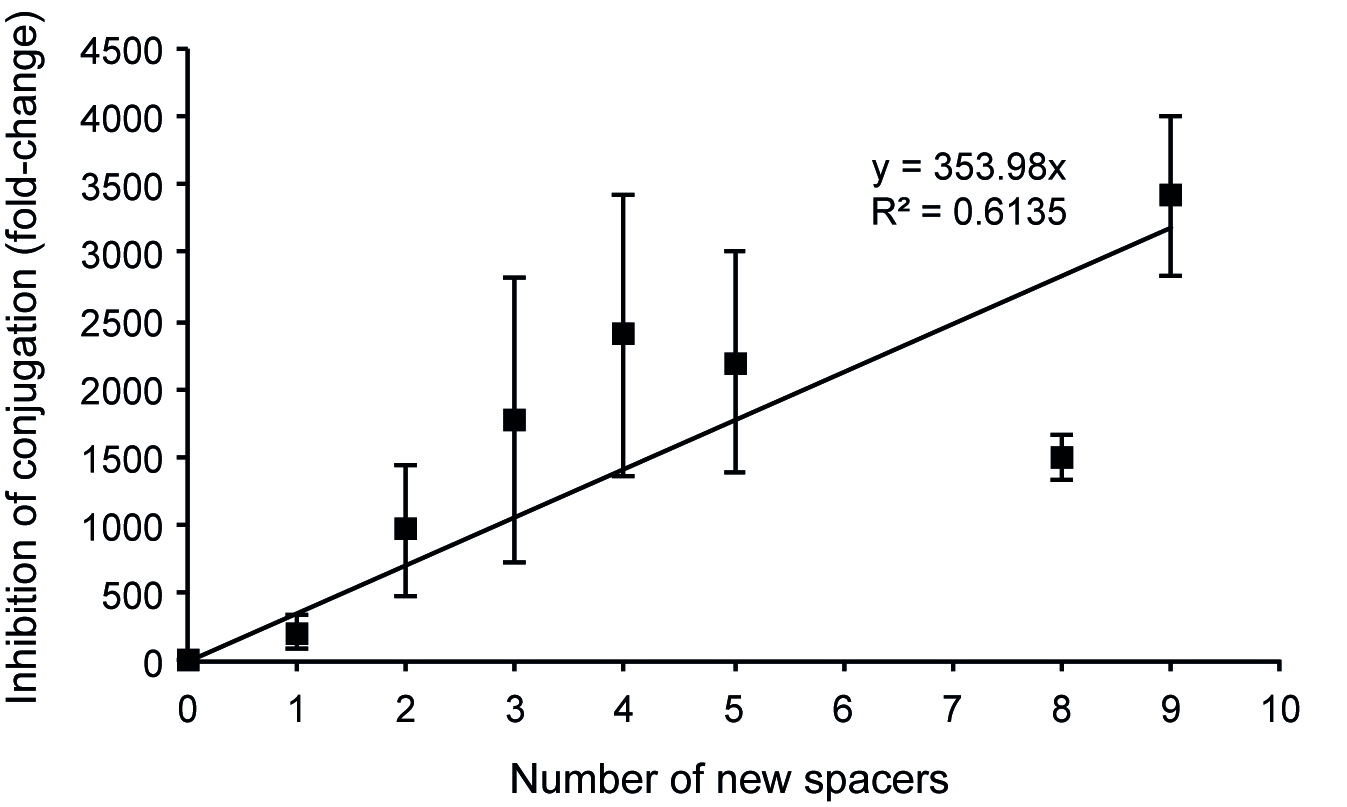


**Figure S2.** Increasing numbers of new spacers increases the resistance to conjugation. Inhibition of conjugation of plasmid pTA142 (*eca0560*) in strains with one (PIM06), two (PIM19), three (PIM13), four (PIM20), five (PIM32), eight (PIM30) or nine (PIM18) new spacers in CRISPR1 or CRISPR2 relative to no new spacers (ΔHAI2). Data shown are the mean ± SD of 3 biological replicates. See also Figure 3.


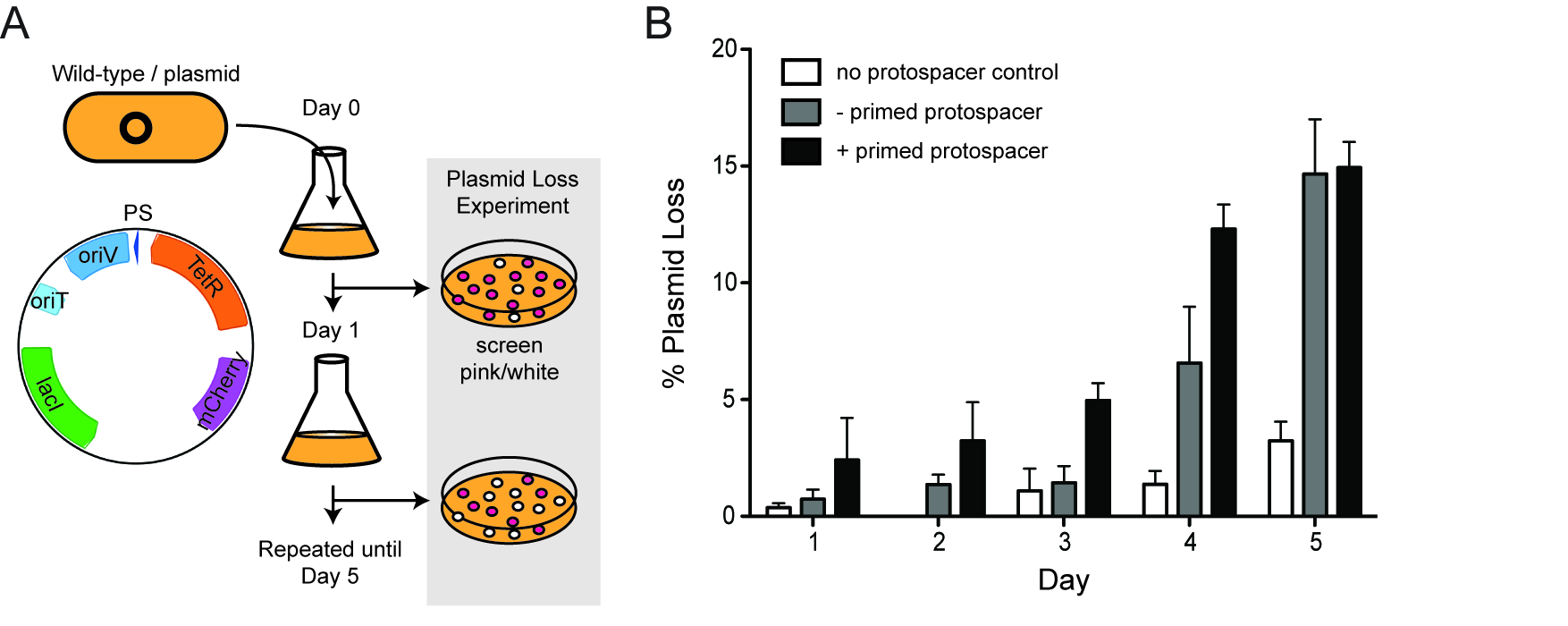


**Figure S3.** (**A**). Schematic of the mCherry plasmid loss assays. *P. atrosepticum* wild-type carrying pPF571 (control), pPF574 (- strand primed protospacer) or pPF575 (+ strand primed protospacer) plasmids were grown without selection for 5 days and (**B**) plasmid loss was scored visually by screening for mCherry activity (pink vs white colonies). Data shown are the mean ± SD of 3 biological replicates.


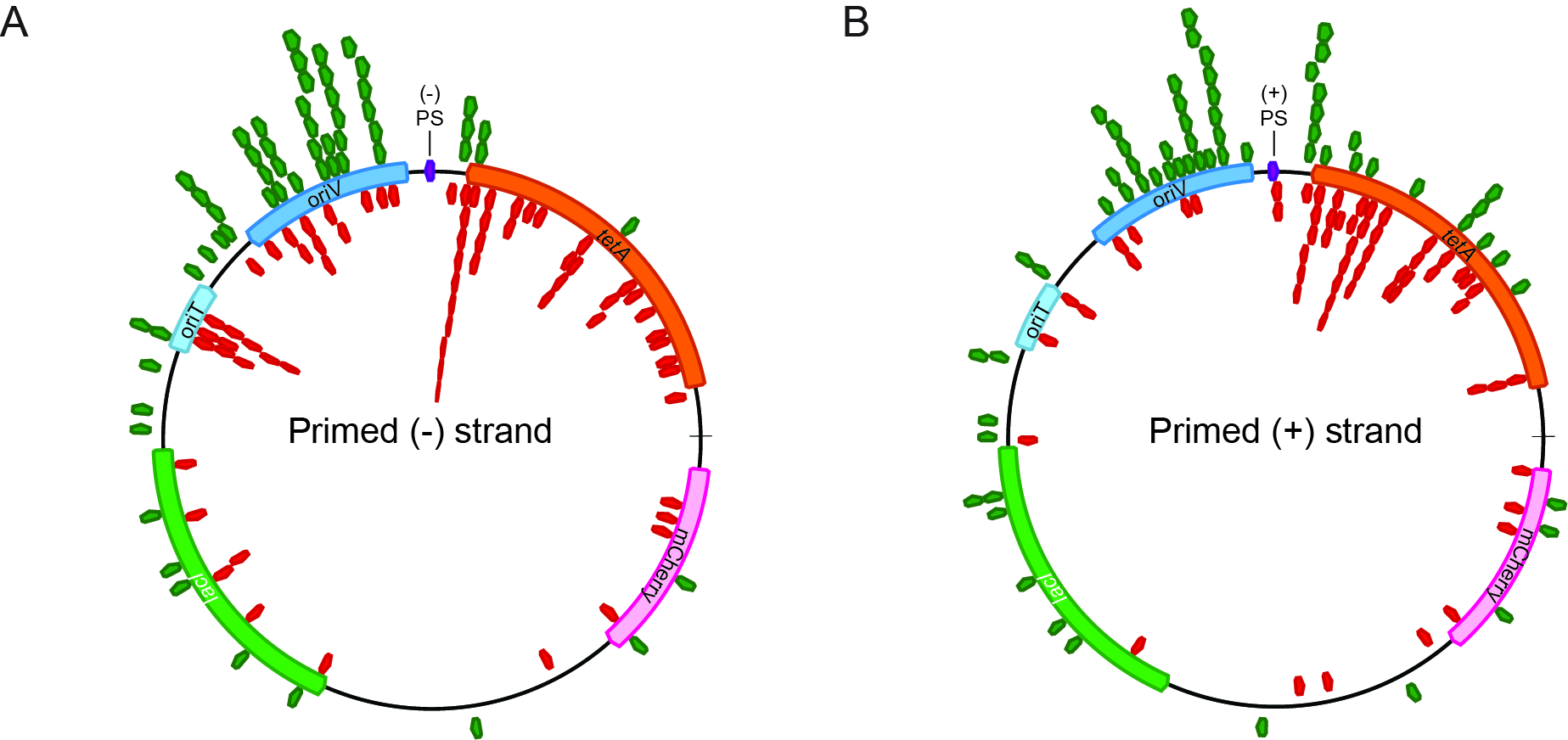


**Figure S4.** Circular maps of the (**A**) – strand primed plasmid (pPF574) and the (**B**) + strand primed plasmid (pPF575) and the location of protospacers targeted by new spacers. The locations of the primed protospacers are indicated with purple triangles. Green triangles represent protospacers on the + strand and red arrows show protospacers on the – strand.


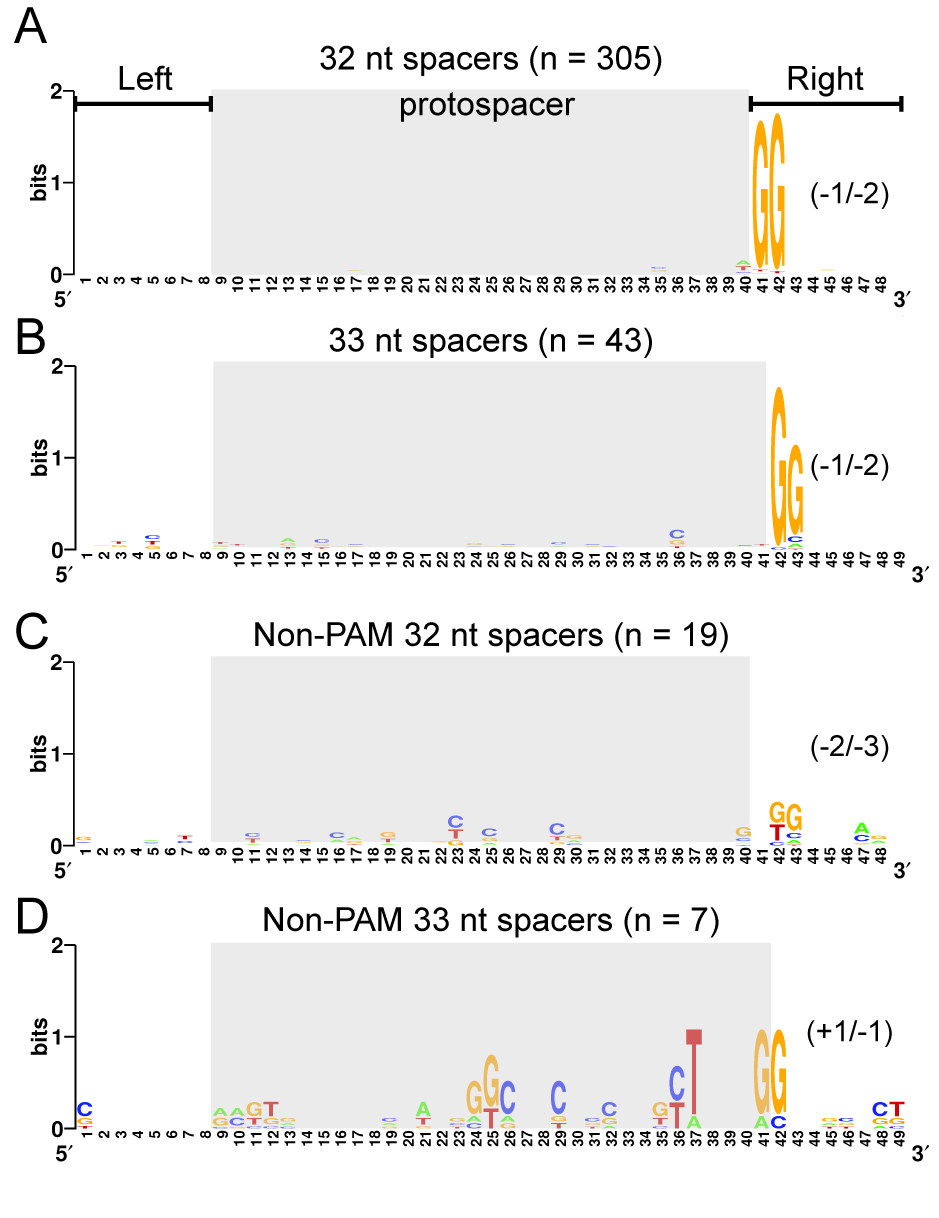


**Figure S5.** PAM analysis. Sequence Logos of protospacers and 8 nt of flanking sequence for spacers of (A) 32 nt, (B) 33 nt and spacer that lack a consensus -1/-2 GG PAM of (C) 32 nt and (D) 33 nt. Sequence Logos were generated at <http://weblogo.berkeley.edu/> ([6](#_ENREF_6)).

**References**

1. Vercoe, R.B., Chang, J.T., Dy, R.L., Taylor, C., Gristwood, T., Clulow, J.S., Richter, C., Przybilski, R., Pitman, A.R. and Fineran, P.C. (2013) Cytotoxic Chromosomal Targeting by CRISPR/Cas Systems Can Reshape Bacterial Genomes and Expel or Remodel Pathogenicity Islands. *PLoS Genet*, **9**, e1003454.

2. Blower, T.R., Evans, T.J., Przybilski, R., Fineran, P.C. and Salmond, G.P. (2012) Viral evasion of a bacterial suicide system by RNA-based molecular mimicry enables infectious altruism. *PLoS Genet*, **8**, e1003023.

3. de Lorenzo, V., Herrero, M., Jakubzik, U. and Timmis, K.N. (1990) Mini-Tn5 transposon derivatives for insertion mutagenesis, promoter probing, and chromosomal insertion of cloned DNA in gram-negative eubacteria. *J Bacteriol*, **172**, 6568-6572.

4. Przybilski, R., Richter, C., Gristwood, T., Clulow, J.S., Vercoe, R.B. and Fineran, P.C. (2011) Csy4 is responsible for CRISPR RNA processing in *Pectobacterium atrosepticum*. *RNA Biol*, **8**, 517-528.

5. Bell, K.S., Sebaihia, M., Pritchard, L., Holden, M.T., Hyman, L.J., Holeva, M.C., Thomson, N.R., Bentley, S.D., Churcher, L.J., Mungall, K. *et al.* (2004) Genome sequence of the enterobacterial phytopathogen *Erwinia carotovora* subsp. *atroseptica* and characterization of virulence factors. *Proc Natl Acad Sci U S A*, **101**, 11105-11110.

6. Crooks, G.E., Hon, G., Chandonia, J.M. and Brenner, S.E. (2004) WebLogo: a sequence logo generator. *Genome Res*, **14**, 1188-1190.
